# Genomic profiling of bacterial and fungal communities and their predictive functionality during pulque fermentation by whole-genome shotgun sequencing

**DOI:** 10.1038/s41598-020-71864-4

**Published:** 2020-09-15

**Authors:** Katherine Chacón-Vargas, Julian Torres, Martha Giles-Gómez, Adelfo Escalante, John G. Gibbons

**Affiliations:** 1 Molecular and Cellular Biology Graduate Program, University of Massachusetts, Amherst, MA 01003 USA; 2 Department of Food Science, University of Massachusetts, Amherst, MA 01003 USA; 3 Departamento de Ingeniería Celular y Biocatálisis, Instituto de Biotecnología, Universidad Nacional Autónoma de México, Cuernavaca, Mexico; 4 Departamento de Biología, Facultad de Química, Universidad Nacional Autónoma de México, Ciudad de México, Mexico; 5 Organismic and Evolutionary Biology Graduate Program, University of Massachusetts, Amherst, MA 01003 USA

**Keywords:** Metagenomics, Microbiology, Food microbiology

## Abstract

Pulque is a culturally important 4,000-year-old traditional Mexican fermented drink. Pulque is produced by adding fresh aguamiel (agave sap) to mature pulque, resulting in a mixture of microbial communities and chemical compositions. We performed shotgun metagenomic sequencing of five stages of pulque fermentation to characterize organismal and functional diversity. We identified 6 genera (*Acinetobacter*, *Lactobacillus*, *Lactococcus*, *Leuconostoc*, *Saccharomyces* and *Zymomonas*) and 10 species (*Acinetobacter boissieri*, *Acinetobacter nectaris*, *Lactobacillus sanfranciscensis, Lactococcus lactis, Lactococcus piscium, Lactococcus plantarum, Leuconostoc citreum, Leuconostoc gelidum, Zymomonas mobilis* and *Saccharomyces cerevisiae*) that were present ≥ 1% in at least one stage of pulque fermentation. The abundance of genera and species changed during fermentation and was associated with a decrease in sucrose and increases in ethanol and lactic acid, suggesting that resource competition shapes organismal diversity. We also predicted functional profiles, based on organismal gene content, for each fermentation stage and identified an abundance of genes associated with the biosynthesis of folate, an essential B-vitamin. Additionally, we investigated the evolutionary relationships of *S. cerevisiae* and *Z. mobilis*, two of the major microbial species found in pulque. For *S. cerevisiae*, we used a metagenomics assembly approach to identify *S. cerevisiae* scaffolds from pulque, and performed phylogenetic analysis of these sequences along with a collection of 158 *S. cerevisiae* strains. This analysis suggests that *S. cerevisiae* from pulque is most closely related to Asian strains isolated from sake and bioethanol. Lastly, we isolated and sequenced the whole-genomes of three strains of *Z. mobilis* from pulque and compared their relationship to seven previously sequenced isolates. Our results suggest pulque strains may represent a distinct lineage of *Z. mobilis*.

## Introduction

Humans have utilized bacteria, yeasts, and molds for millennia in the production of traditionally fermented foods and beverages^1,2^. Microbial fermentation was a key innovation as it played a crucial role in improving food preservation, nutritional content, consistency, and flavor^1,3^. Fermented foods are typically produced by a community of microbes that fluctuate in abundance and diversity during the fermentation process^4–9^. The traditional artisanal practice of backslopping, the addition of a small quantity of a mature fermented food to the beginning of a new fermentation, has led to the reproducible formation of these beneficial microbial communities, and their associated metabolic transformations of food and beverages^1,2,10^.

Pulque is a traditional Mexican alcoholic beverage produced by fermenting aguamiel (agave sap)^11,12^. Archaeological chemistry evidence suggests that the origins of pulque date to at least 1,500 years ago^13^, and historical documents suggest that pulque was being produced as far as 4,000 years ago^11,12^. Pulque has served as a sacred beverage during religious ritual, is heralded as a nutritionally-rich supplement, and is a source of cultural pride and identity^11,12^. Modern production of pulque has remained nearly identical to production during pre-Hispanic times^11^. Briefly, aguamiel is extracted from mature agave plants, this fresh collected aguamiel is mixed with mature pulque, and the mixture is then fermented in vats from three hours to as long as 12 days^11^.

Pulque fermentation is carried out by a suite of microorganisms that produce three metabolites distinctive to pulque^11,14^. Lactic acid bacteria and acetic acid bacteria produce the characteristic acidity, which ranges from a pH of 3.5–4.2^11^. The ethanol content of pulque, which ranges between 4 and 7%, is produced through the metabolism of sugars by yeasts (*Saccharomyces* sp., *Kluyveromyces* sp. etc*.)* and the bacteria *Zymomonas mobilis*^11,15^. Lastly, species from the lactic acid bacteria genus *Leuconostoc* produce extracellular polysaccharides (EPS) resulting in pulque’s distinctive viscosity^16^. Lactic acid bacteria and *S. cerevisiae* have been isolated from aguamiel^17–19^, which likely represent the major source of microorganisms for pulque fermentation. The source of *Z. mobilis* in pulque is less clear, as the specie has not been directly isolated from or detected in aguamiel. However, aguamiel’s high sugar content suggests that it is a suitable natural habitat^20,21^. Alternatively, the identification of *Z. mobilis* from honey bees^22^ and the practice of filtering aguamiel to remove insects and other debris suggests a possible insect transmission^11^.

Studies focused on the microbial diversity of pulque have used traditional culture-based approaches^14,15,23^, and more recently, high-throughput 16S/ITS rDNA metagenomics sequencing^24,25^. However, whole-genome shotgun metagenomic sequencing has not been applied to study the microbial and metabolic diversity of pulque fermentation. The advantages of shotgun metagenomic sequencing over amplicon sequencing include (i) the absence of amplification bias, (ii) the joint and comprehensive examination of the entire microbial community (bacterial and fungal), and (iii) the ability to draw functional inferences from gene abundance data^26,27^. Here, we conducted shotgun metagenomic sequencing across five distinct stages of pulque fermentation to investigate microbial and functional diversity.

## Results

### Metagenomics DNA extraction and sequencing of pulque fermentation

We extracted metagenomic DNA of four technical replicates from fresh aguamiel (AM), a mixture of fresh AM with mature pulque (0 h and start of fermentation, T0), 3 h into fermentation (T3), 6 h into fermentation (T6), and 12 h into fermentation (mature pulque, PQ). Our DNA extraction protocol yielded high molecular weight DNA suitable for shotgun metagenomics sequencing (Supplementary Figure S1). As a quality control step to demonstrate the integrity of metagenomic DNA extraction, we successfully amplified the 16S rDNA (V3–V4) locus in each of the technical replicates (Supplementary Figure S2). We generated a total of 18,750,182, 7,798,353, 17,691,690, 20,946,800, and 25,133,620 read pairs across the technical replicates for the AM, T0, T3, T6, and PQ stages, respectively. Species abundance between technical replicates was highly correlated (all comparisons *r* > 0.99). Thus, we combined technical replicate data for each fermentation stage for subsequent analysis.

### Taxonomic profiling of microbial community during pulque fermentation

Taxonomic profile and relative abundance of the microbial community was assessed during stages of pre-fermentation (AM) and fermentation (T0, T3, T6, and PQ). We used MetaPhlAn^28^ and Kaiju^29^ for taxonomic classification. Both methods were highly agreeable at the genus- and species-levels (Supplementary Tables S1–4). For instance, all of the major 14 genera identified with MetaPhlan were also identified with Kaiju, and Pearson correlations (*r*) of percent genus abundance across fermentation stages between the two methods averaged 0.70. Considering the strong agreement between methods, we primarily report results from Kaiju because it uses a larger database for taxonomic classification and thus, has increased resolution at the species-level^29^.

Viruses and archaea made up less than 0.4% and 0.2% of assigned reads across all stages of pulque fermentation. Bacteria made up 99%, 85%, 75%, 92%, and 82% and fungi made up 0.6%, 14%, 25%, 8%, and 18% of assigned reads across the AM, T0, T3, T6, and PQ stages, respectively. Six, 8, and 31 genera and 10, 15, and 56 species were present ≥ 1%, ≥ 0.5%, and ≥ 0.1% in at least one fermentation stage, respectively (Supplementary Tables 3 and 5). Six of the 56 species present ≥ 0.1% in at least one fermentation stage were fungal (*Kluyveromyces marxianus*, *Saccharomyces arboricola*, *S. cerevisiae*, *S. cerevisiae* x *Saccharomyces kudriavzevii*, *Saccharomyces eubayanus* and *S. kudriavzevii*).

**Figure 1:**
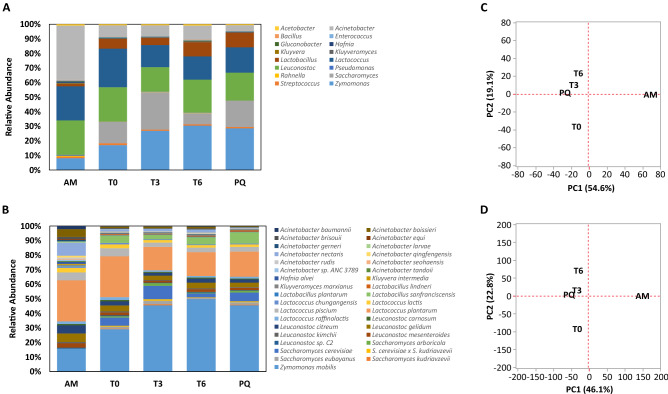
Organismal diversity during pulque fermentation. (**A**) Genus-level and (**B**) Species-level relative abundance during pulque fermentation of organisms present ≥ 0.2% in at least one fermentation stage as estimated by Kaiju^29^. Columns represent relative abundance of organisms (Y-axis) per fermentation stage (X-axis). AM = Aguamiel, T0 = pulque and aguamiel mixture, T3 = 3-h fermentation, T6 = 6-h fermentation, and PQ =  ~12-h fermentation (mature pulque). Values are reported as percentages. Principal component analysis (PCA) of relative organismal abundance for genera (**C**) and species (**D**). In both comparisons, principal components 1 and 2 explain > 68% of variance.

Overall, AM showed the highest diversity (Simpson’s diversity (*D*) and Shannon’s diversity (*H*)) (*D*_AM_ = 9.03, *H*_AM_ = 2.86), followed by T0 (*D*_T0_ = 5.88, *H*_T0_ = 2.43), while the T3, T6, and PQ stages showed lower levels of diversity (*D*_T3_ = 4.19, *H*_T3_ = 2.21, *D*_T6_ = 3.71, *H*_T6_ = 2.15 and *D*_PQ_ = 4.10, *H*_PQ_ = 2.13). At the genus-level, AM was dominated by *Acinetobacter* (21.95%), *Leuconostoc* (13.92%), *Lactococcus* (13.72%), *Zymomonas* (4.77%) and *Lactobacillus* (0.97%), and the most abundant species were *Lactococcus plantarum* (8.50%), *Z. mobilis* (4.78%), *Acinetobacter nectaris* (2.68%), *Leuconostoc gelidum* (1.77%), *Leuconostoc citreum* (1.68%), *Leuconostoc piscium* (1.65%), *Acinetobacter boissieri* (1.59%) and *Lactococcus lactis* (0.97%) (Fig. 1, Supplementary Tables S2 and S4). The most abundant genera during the T0 stage was *Lactococcus* (19.52%), *Leuconostoc* (17.34%), *Zymomonas* (12.57%), *Saccharomyces* (10.79%), *Acinetobacter* (5.73%) and *Lactobacillus* (4.94%) and the most abundant species were *Z. mobilis* (12.57%), *L. plantarum* (12.11%), *L. piscium* (2.44%), *Saccharomyces cerevisiae* (2.42%), *Lactobacillus sanfranciscensis* (2.22%), *L. gelidum* (1.77%), *L. citreum* (1.68%), and *L. lactis* (0.97%) (Fig. 1, Supplementary Tables S2 and S4). The T3 stage was dominated by the genera *Zymomonas* (19.91%), *Saccharomyces* (19.08%), *Leuconostoc* (12.38%), *Lactococcus* (11.25%), *Acinetobacter* (5.45%) and *Lactobacillus* (3.61%) and the species *Z. mobilis* (19.91.2%), *L. plantarum* (7.08%), *S. cerevisiae* (4.05%), *Lactobacillus sanfranciscensis* (1.41%), *L. piscium* (1.31%), *L. gelidum* (1.29%), and *Saccharomyces eubayanus* (0.99%) (Fig. 1, Supplementary Tables S2 and S4). The most abundant genera during the T6 stage were *Zymomonas* (22.27%), *Leuconostoc* (16.50%), *Lactococcus* (11.73%), *Acinetobacter* (7.17%), *Lactobacillus* (7.08%), and *Saccharomyces* (5.65%) and the most abundant species were *Z. mobilis* (22.27%), *L. plantarum* (7.33%), *L. sanfranciscensis* (2.20%), *L. gelidum* (1.72%), *L. piscium* (1.39%), *S. cerevisiae* (1.25%), and *L. citreum* (1.20%) (Fig. 1, Supplementary Tables S2 and S4). Finally, the most abundant genera in the PQ stage were *Zymomonas* (21.48%), *Leuconostoc* (14.30%), *Saccharomyces* (13.51%), *Lactococcus* (13.03%), *Lactobacillus* (7.53%), and *Acinetobacter* (2.85%) and the species *Z. mobilis* (21.48%), *Lactococcus plantarum* (8.11%), *L. sanfranciscensis* (3.71%), *S. cerevisiae* (2.86%), *L. piscium* (1.57%), *L. gelidum* (1.47%) and *L. citreum* (1.04%) (Fig. 1, Supplementary Tables S2 and S4). Principal Component Analysis of relative organismal abundance at the genus and species level showed nearly identical patterns and clear separation between AM from all fermentation stages as well as separation between T0 from T3, T6 and PQ (Fig. 1C, D, Supplementary Figure S3).

**Figure 2:**
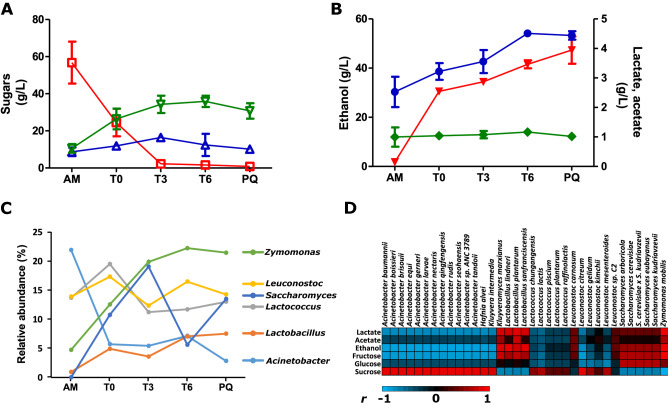
Temporal patterns of chemical and organismal abundance during pulque fermentation. (**A**) Concentration of the main sugars and (**B**) ethanol, lactic and acetic acid (**B**) during pulque fermentation (red square—sucrose, blue triangle—glucose, green down triangle—fructose, red filled down triangle—ethanol, green filled diamond—acetic acid, blue filled circle—lactic acid). (**C**) Relative abundance of the dominating genera (Y-axis) during pulque fermentation (X-axis). AM = Aguamiel, T0 = pulque and aguamiel mixture, T3 = 3-h fermentation, T6 = 6-h fermentation, and PQ =  ~ 12 h fermentation (mature pulque). (**D**) Heat map of Pearson correlations (*r*) between species abundance for organisms present ≥ 0.2% in at least one fermentation stage and chemical concentrations across pulque fermentation.

Next, we evaluated the temporal patterns of the major genera and species (present ≥ 1% in at least one fermentation stage) and their associations with the main fermentative products of pulque^11^ (i.e. acetic acid, lactic acid, ethanol, and polysaccharides from available sugars in AM). Pearson correlation values for all combinations of metabolites and the 35 species present ≥ 0.1% in at least one fermentation stage are reported in Supplementary Table S5. *Acinetobacter, Lactococcus* and *Leuconostoc* were highly abundant in the AM stage (> 13%). Both *Lactococcus* and *Leuconostoc* fluctuated slightly during fermentation but were present at approximately the same abundance in PQ (Fig. 2). Conversely, *Acinetobacter* decreased sharply after AM, eventually reaching 2.85% in PQ (Fig. 2A). *Acinetobacter* abundance was positively associated with sucrose (*r* = 0.91, *p* value = 0.031) and negatively associated with fructose (*r* =  −0.89, *p* value = 0.046) and ethanol (*r* =  −0.93, *p* value = 0.022) (Fig. 2). Ten species of *Acinetobacter* displayed identical significant negative associations with ethanol and fructose, and significant positive associations with sucrose Supplementary Table S5. *Zymomonas, Saccharomyces,* and *Lactobacillus* were all present < 4% in AM, but by the end of fermentation (PQ) reached 21.48%, 13.51% and 7.53%, respectively (Fig. 2). Notably, the increase in relative abundance of *Zymomonas* was positively correlated with the increased production of ethanol (*r* = 0.95, *p* value = 0.014), fructose (*r* = 0.96, *p* value = 0.010) and lactate (*r* = 0.95, *p* value = 0.0143), and negatively correlated with sucrose (*r* =  −0.99, *p* value = 0.0016) (Fig. 2). *Zymomonas* and *Z. mobilis* were the only genus or species significantly positively correlated with fructose (Supplementary Table S5). The increase in relative abundance in *Lactobacillus* was positively correlated with ethanol (*r* = 0.95, *p* value = 0.0137) and lactate (*r* = 0.95, *p* value = 0.0128). *Saccharomyces* was at low relative abundance during AM (0.033%) and fluctuated during fermentation until reaching 13.51% in PQ. Interestingly, *Saccharomyces* abundance was not significantly correlated with sugar or ethanol abundance, reinforcing the role of *Zymomonas* in ethanol production during pulque fermentation. At the species-level, the yeast *Kluyveromyces marxianus* was also significantly positively associated with ethanol abundance (*r* = 0.88, *p* value = 0.049) (Supplementary Table S5).

### Functional profiling during pulque fermentation

**Figure 3:**
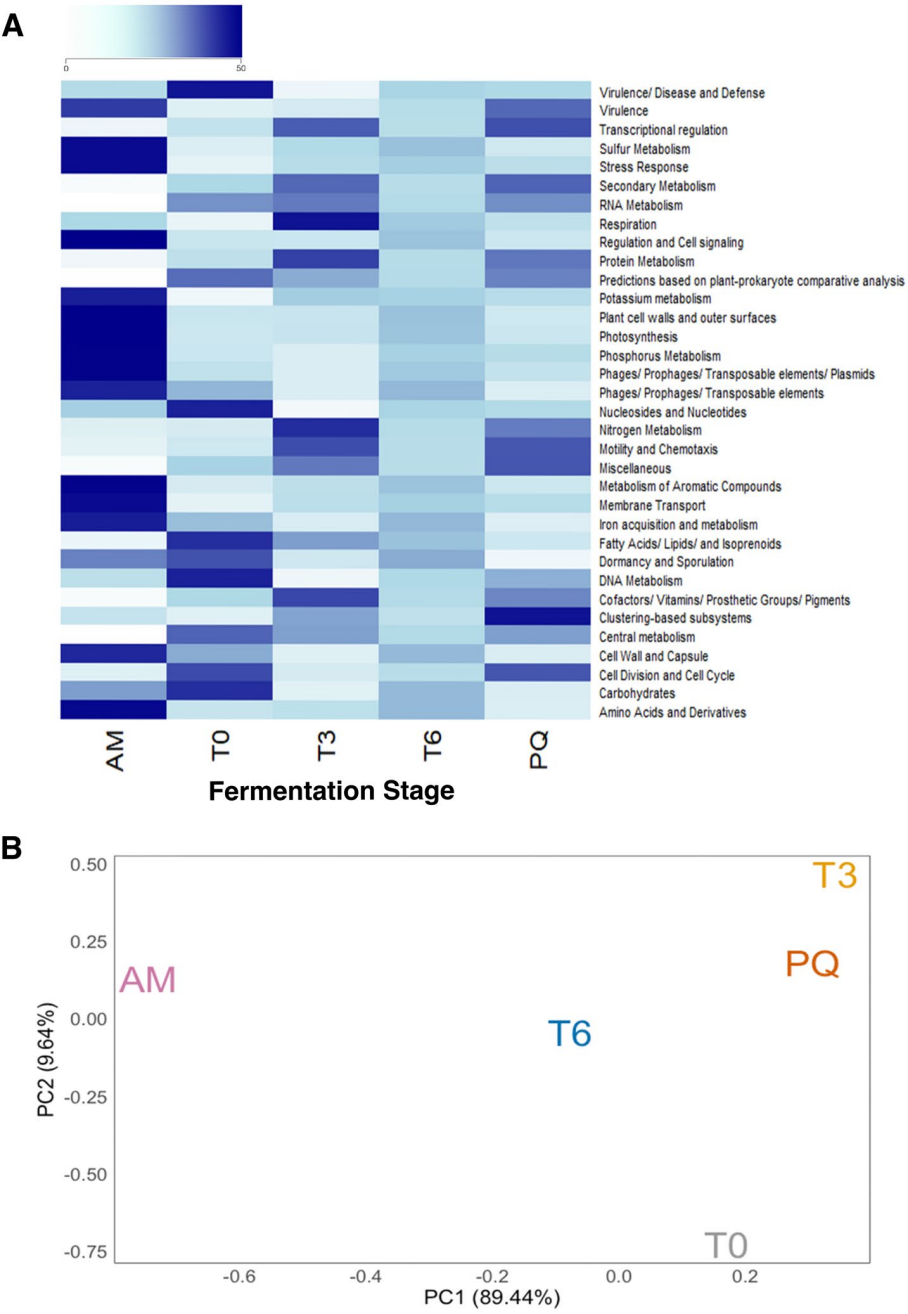
Metagenomic functional profiles change during pulque fermentation. (**A**) Heatmap of relative abundance for level-1 functional groups as defined by SUPER-FOCUS^30^ (X-axis) during pulque fermentation (Y-axis), where dark blue indicates high abundance and light blue indicates low abundance. (**B**) Principal Component Analysis (PCA) of functional profiles. Principal components 1 and 2 explain > 99% of variance.

We used the Super-Focus pipeline to evaluate the functional profile of each stage^30^. We identified 34, 183, 1,153, and 17,544 functional clusters for level 1, level 2, level 3, and the seed level, respectively (Fig. 3A, Supplementary Figure S4A). Consistent with the taxon abundance profiles (Fig. 1), PCA of functional cluster relative abundance showed clear separation between AM to all fermentation stages (Fig. 3B, Supplementary Fig. 4B).

**Figure 4:**
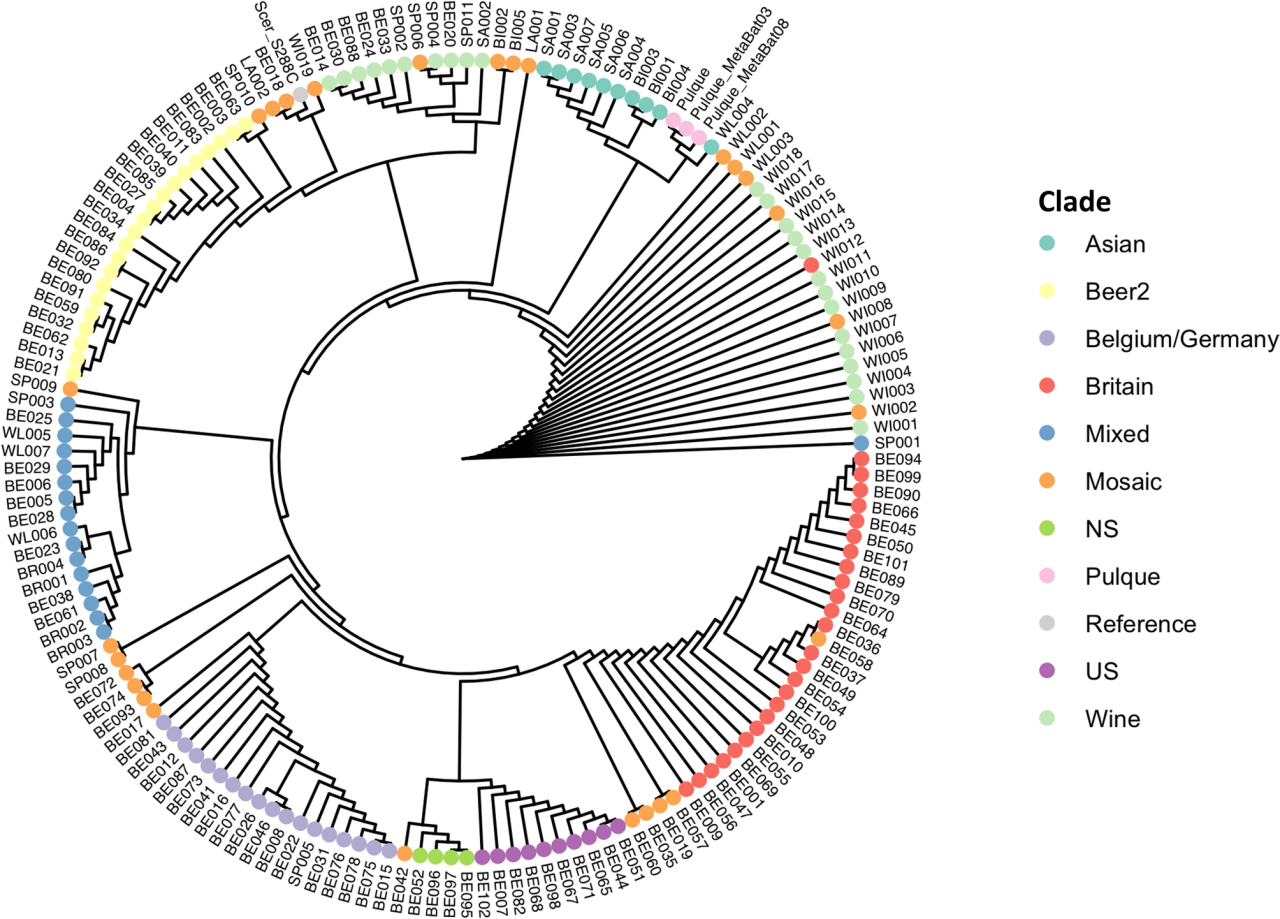
Evolutionary relationship of *Saccharomyces cerevisiae* metagenomics assembly from pulque and isolates from fermented beverages. Phylogenetic tree of 157 isolates from Gallone et al.^31^, and the S288C reference genome with translated CDS sequences predicted from scaffolds assigned as *S. cerevisiae* using Autometa (Pulque) and MetaBat2 (Pulque_MetaBat03 and Pulque_MetaBat08). Clades are labeled as in Gallone et al.^31^. The *S. cerevisiae* metagenomics assembly from pulque is nested within the “Asian” clade.

Next, we compared the functional profiles of AM versus PQ, and each sequential stage of fermentation (AM vs. T0, T0 vs. T3, T3 vs. T6, and T6 vs. PQ). Presence/absence data for each Super-Focus subsystem across 26 of the 29 bacterial genera present in the Super-Focus database are reported in Supplementary Data S1. For level-2, there were 85 functional groups that displayed significant differences in abundance between AM versus PQ, representing pre-fermentation and final fermentation (Supplementary Table S6). Sixty-three of the significant functional groups were more abundant in AM and 22 functional groups were more abundant in PQ. Interestingly, many of the functional groups enriched in AM were associated with bacterial defense and stress response (e.g. “Bacteriocins, Ribosomally Synthesized Antibacterial Peptides”, “General Stress Response and Stationary Phase Response”, “Osmotic Stress”, “Pathogenicity Islands”, “Phages, Prophages”, “Programmed Cell Death and Toxin-antitoxin Systems”, “Transposable Elements”, “Bacteriophage integration/excision/lysogeny” etc*.*). Functional groups enriched in PQ included “Carotenoid Biosynthesis”, “Folate and Pterines”, and “Proteolytic Pathway” (Supplementary Table S6).

AM versus T0 compares fresh aguamiel to the backslopping stage, where fresh aguamiel is added to a container with fermented pulque (in the vessel where fermentation takes place). The T0 stage introduces water, sugars (mainly sucrose) (Fig. 2B) and microorganisms present in the fresh aguamiel. Between AM and T0 we identified 71 functional groups in level-2 that were significantly different in abundance. Fifty-four functional groups had a high abundance in AM and 17 had a high abundance in T0 (Supplementary Table S7). Several of the functional groups that were more abundant in AM were associated with primary metabolism, bacterial defense and stress response (e.g. “A bicyclomycin resistance protein, a helicase, and a pseudouridine synthase “, “Fatty Acid Metabolic Cluster”, “General Stress Response and Stationary Phase Response”, “Pathogenicity Islands”, “Programmed Cell Death and Toxin-antitoxin Systems”, “Shiga Toxin Cluster”, “Transposable Elements”). Functional groups with elevated abundance in T0 included “Carotenoid Biosynthesis”, “Proteolytic Pathway”, “Biosynthesis of Phenylpropanoids” and “Lactate Racemization” (Supplementary Table S7).

The T0 versus T3 comparison revealed 27 functional groups with differential abundance, with 14 and 13 at higher abundance in T0 and T3, respectively (Supplementary Table S8S8). Some of the functional groups enhanced in T0 were “Gram-positive Cell Wall Components”, “Acid Stress” and “Bacterial Checkpoint Control”. In T3, we observed elevated abundance of the functional groups “Carotenoid Biosynthesis”, “Inorganic Sulfur assimilation”, and “Proteolytic Pathway”. The T3 versus T6 comparison revealed only 12 functional groups with differential abundance, with 8 and 4 groups displaying elevated abundance in T3 and T6, respectively. Functional groups with higher abundance in T3 included “Polysaccharides” and “Proteolytic Pathway” while functional groups higher in abundance in T6 included “Invasion and Intracellular Resistance” and “Lactate Racemization” (Supplementary Table S9). Finally, 28 functional groups showed differential abundance in T6 versus PQ. We found 24 functional groups that displayed elevated abundance in T6 and 4 functional groups that displayed elevated abundance in PQ. Functional groups elevated in T6 included “Programmed Cell Death and Toxin-antitoxin Systems”, “Metabolism of Central Aromatic Intermediates”, “Lactate racemization”, “Triacylglycerols”. Functional groups elevated in PQ included “Proteolytic pathway”, “Toxins and Superantigens”, “Nucleotide Sugars” and “Plant Alkaloids” (Supplementary Table S10).

### Evolutionary relationship of metagenomics assembly of *S. cerevisiae* from pulque

Because *S. cerevisiae* is a prominent species found in many fermented foods and beverages, and *S. cerevisiae* evolutionary history is well-studied^31,32^, we aimed to assess how *S. cerevisiae* from pulque is related to other strains isolated from fermentations and other sources. We performed a metagenomics assembly of Illumina reads from PQ using metaSpades^33^ in order to identify and extract scaffolds assigned to *S. cerevisiae*. Using Autometa^34^ and Metabat^35^, we identified three bins containing *S. cerevisiae* scaffolds. We recovered 368, 190, and 184 scaffolds totaling 5.07 Mb (Pulque_Metabat03), 5.19 Mb (Pulque_Metabat08), and 3.50 Mb (Pulque_Autometa) in which we predicted 2,753, 2,805, and 1,922 protein-coding genes.

We analyzed the *S. cerevisiae* pulque metagenomic assembly data with 157 *S. cerevisiae* strains from Gallone et al*.*^31^, and the SC288 reference genome^36^. We used OrthoFinder^37^ to identify 7,232 ortholog groups. This set of translated coding sequences were used to construct a phylogenetic tree using the Species Tree from All Genes algorithm (STAG)^38^. Importantly, this analysis was highly agreeable with results from Gallone et al.^31^, and placed the majority of isolates into their major lineages (Fig. 4). The *S. cerevisiae* metagenomic assembly was positioned within the Asian clade with samples isolated from sake and bio-ethanol (Fig. 4).

### Evolutionary relationship of *Z. mobilis* strains isolated from pulque

**Figure 5:**
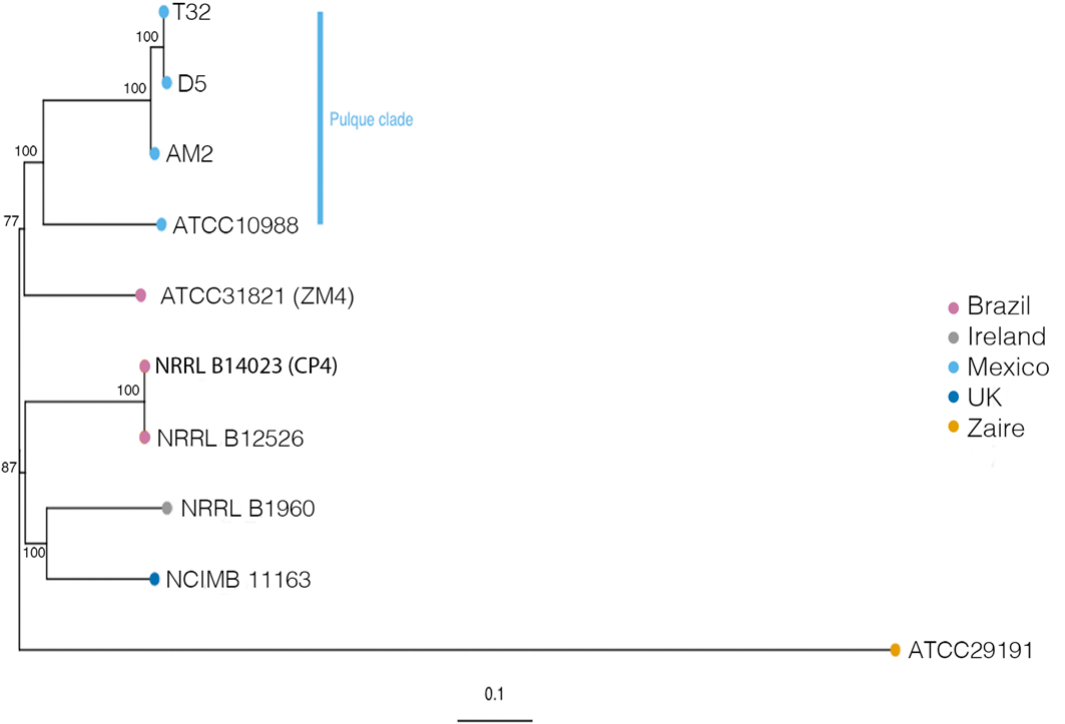
Evolutionary relationship of *Zymomonas mobilis* isolates. Maximum likelihood phylogenetic tree of *Z. mobilis* isolates with complete genomes or whole-genome sequence data reconstructed from 74,825 genome-wide SNPs using PhaME pipeline^39^. Bootstrap values are reported for each node. Strains T32, D5, AM2 and ATCC10988 were isolated from pulque and are monophyletic.

We isolated three strains of *Z. mobilis* from pulque from Huitzilac, a town in Morelos State, Mexico (altitude of 2,550 m in a cold weather mountainous region) and sequenced their genomes in order to determine their evolutionary relationships to other sequenced strains. The batch of pulque from which these strains were isolated from was used in the T0 backslopping stage of our experiment, when fresh aguamiel was mixed with mature pulque. Cumulative genome assembly size ranged from 2.07 to 2.49 Mb, with GC content ranging from 32 to 36%. Using the PhAME pipeline^39^, we identified 74,825 polymorphic sites between the genomes of the three *Z. mobilis* strains from pulque and an additional 7 previously sequenced *Z. mobilis* genomes^40–45^. Phylogenetic analysis revealed that the pulque isolates we sequenced were monophyletic with a previously sequenced strain ATCC 10988, which was also isolated from pulque (Fig. 5). This relationship was supported with 100% bootstrap support. The pulque clade showed a close proximity with strain ATCC 31821 from Brazil that was originally isolated from sugarcane^45^. Strains NCIMB 11163 from England and strain NRRL B-1960 from Scotland isolated from beer and cider, respectively, were clustered together. Strains NRRL-B-14023 and NRRL-B-12526, which are clones, composed another group. Strain ATCC 29191 isolated from palm sap in Zaire displayed the most divergent patterns of polymorphism. Phylogenetic network analysis reinforced these relationships (Supplementary Figure S5), as well as the results of a previous phylogenetic analysis of a subset of these strains^46^.

### Presence of *Z. mobilis* strains isolated from pulque across fermentation

We assessed the relatively proportion of the sequenced *Z. mobilis* isolates across fermentation. We carried out two independent methods to identify and quantify the relative abundance of the AM2 and D5/T32 *Z. mobilis* genotypes from our metagenomics data. D5 the T32 isolates are very closely related and may represent a clonal lineage or the same isolate (Fig. 5). Thus, we grouped these isolates together. First, we identified 380 single nucleotide polymorphisms (SNPs) that differentiate the AM2 and D5/T32 genotypes. We then calculated allele frequency for each locus across fermentation stages. The averaged allele frequencies suggests that the relative frequency of AM2 genotype to the D5/T32 genotype ranges from 67 to 73% (Supplementary Figure S6A). Additionally, we identified lineage specific genes between the AM2 and D5/T32 genotypes and calculated their average coverage across fermentation stages. The trend of this analysis agrees with the SNP analysis, and suggests that the relative frequency of the AM2 genotype ranges from 80%-88%, while the D5/T32 genotype ranges from 12 to 20% (Supplementary Figure S6B). Taken together, our results suggests that the *Z. mobilis* genotypes were relatively stable across fermentation and that the AM2 genotype was more prevalent in our fermentation than the D5/T32 genotype.

## Discussion

We used shotgun metagenomics sequencing to analyze the microbial and functional diversity during pulque fermentation, and metagenomic assembly and whole-genome sequencing to investigate the relationship of *S. cerevisiae* and *Z. mobilis* strains from pulque, respectively. These analyses yielded several key findings, which collectively shed light on microbial and functional diversity during pulque fermentation. First, we provide the first direct evidence of *Z. mobilis* in aguamiel (Figs. 1, 5). Although aguamiel has been a suspected reservoir of *Z. mobilis*, isolation and detection have remained elusive^17,21,24,25,47^. For instance, *Z. mobilis* was not detected in aguamiel from *Agave atrovirens* and *Agave salmiana* across four seasons using 16S rDNA sequencing and denaturing gradient gel electrophoresis^24^, or from aguamiel from three different locations in Hidalgo State using high-throughput 16S rDNA amplicon sequencing^25^. However, *Z. mobilis* was identified from aguamiel using selective media, though without validation of species identity with 16S rDNA sequencing^12^. These results suggest that the presence of *Z. mobilis* in aguamiel may be variable, and perhaps dependent on a number of factors including agave species, geography, environmental conditions etc*.*

We identified a cohesive set of organisms that were consistently present across most or all stages of pulque fermentation. These organisms included several species of lactic acid bacteria, acetic acid bacteria, *Z. mobilis*, and *S. cerevisiae* (Figs. 1, 2C, D). Interestingly, the acetic acid bacteria genus *Acinetobacter* made up the highest percentage of organisms in aguamiel (when sucrose concentration is highest), followed by *Leuconostoc* and *Lactococcus* (Figs. 1, 2). The abundance of *Acinetobacter* dropped sharply from 22 to ~ 2.8% during fermentation, while the lactic acid bacteria genera *Leuconostoc* and *Lactococcus* remained relatively stable across fermentation (Figs. 1, 2). Importantly, these genera have been found in high abundance in aguamiel and pulque^23–25,48^. Temporal shifts in microbial composition during fermentation are consistent with most traditionally fermented foods and beverages^4,10^. In pulque, shifts in microbial abundance likely reflect changes in resource competition (e.g. sucrose consumption, acid and alcohol production and tolerance etc.) (Fig. 2A, B). For example, strains of *S. cerevisiae* isolated from pulque show higher levels of ethanol tolerance than strains isolated from aguamiel where ethanol content is lower^18^.

In pulque, the native microbial community is likely related to the traditional non-aseptic conditions during the collection, transportation, and fermentation of aguamiel^11,49^. For instance, the identification of *Z. mobilis* from honeybees^22^ and the practice of filtering aguamiel to remove insects and other debris^11^ suggests a possible insect transmission of microbial community members. Consistent with these fermentation practices, our analysis showed that the aguamiel stage differed most compared with the other fermentation stages, and was the most diverse (Fig. 1C, D). It is clear that the microbial community of aguamiel is variable^24^, and these differences could translate into regional, seasonal, and environmental differences in the microbial communities of pulque.

There is a long history reporting the nutritional benefits associated with low-level consumption of pulque. For instance, pulque is a major source of vitamin C, and was used to treat scurvy in Mexican penitentiary inmates in the late 1800s^11^. Supporting this observation, more recently, a study of dietary patterns in rural central Mexico revealed that pulque is the most important source of ascorbic acid^50^. Another early study of pulque from 1946 in the indigenous Otomí population in Hidalgo state demonstrated that pulque was the second most important food in the diet after tortilla, because it provided substantial amounts of calories, total protein, thiamin, riboflavin, niacin, vitamin C, calcium, and iron^11^. More recent analysis supports the importance of pulque as a dietary source of iron and folates^50,51^. Folic acid deficiency during pregnancy can lead to neural tube defects^52^, and, importantly, pulque intake is a strong indicator of folate status in rural Central Mexican populations^50^. Our functional enrichment analysis suggests that microbial genes involved in folate biosynthesis are present in all major bacterial genera and significantly more abundant in pulque than in aguamiel (Supplementary Table S6, Supplementary Data S1). However, it is important to note that while low or moderate pulque intake during pregnancy and lactation may have some positive health benefits, heavy pulque intake during lactation is significantly associated with adverse postnatal growth^53^.

Though pulque fermentation was dominated by bacteria, we identified 6 fungal species that were present ≥ 1% in at least one stage of fermentation (Fig. 1, Supplementary Table S4). In agreement with previous work^23^, we observed a drastic increase in yeast abundance when mature pulque was mixed with fresh aguamiel (T0 stage) (Figs. 1, 2). *S. cerevisiae* was the most abundant species at each stage, but *S. eubayanus*, *S. arboricola*, *S. kudriavzevii*, and *S. cerevisiae* x *S. kudriavzevii* hybrids were all detected ≥ 0.1% during all stages of fermentation with the exception of AM. Interestingly, *S. eubayanus* and *S. kudriavzevii* are both cryotolerant species^54,55^. The thermotolerant yeast *K. marxianus* was also present at ≥ 0.1% during all stages of fermentation. *K. marxianus* has been previously identified from aguamiel and pulque^18,25,56,57^, as well as from agave used to ferment other traditionally distilled beverages^15^, and can ferment a more diverse and complex set of substrates than *Saccharomyces* species^58–61^. The observation that *Saccahromyces* species were not significantly correlated with ethanol production, but *K. marxianus* was, may suggest unequal roles for yeasts in alcohol production during pulque fermentation.

Lastly, because *Z. mobilis* is the dominant species in pulque and contributes to the alcohol content of pulque^11,14,23^, we isolated three *Z. mobilis* strains from pulque to understand their evolutionary relationship with previously sequenced isolates. Using maximum likelihood phylogenetic analysis and phylogenetic network analysis of ~ 74825 SNPs scattered across the genome, our results show a distinct “pulque” clade made up of the three strains sequenced here, and a previously sequenced strain isolated from pulque (ATCC 10988) (Fig. 5, Supplementary Figure S5). The presence of monophyletic groups from particular fermented food sources is indicative of microbial domestication^31,62,63^. However, we acknowledge that our results could be the outcome of sampling bias. Assessing whether *Z. mobilis* strains isolated from pulque represent a domesticated lineage will require more extensive sampling of pulque and environmental strains across Mexico and globally, as well as phenotypic work to asses characteristics unique to pulque derived isolates.

## Materials and methods

### Laboratory pulque fermentation

Pulque (PQ) (approximately 12 h of overnight fermentation) and fresh extracted aguamiel (AM) samples were collected from the town of Huitzilac, Morelos State, Mexico (altitude of 2,550 m in a cold weather mountainous region, 19°01′42″N 99°16′02″W). Samples were placed in sterile plastic bags and transported immediately to the laboratory. A laboratory fermentation was carried out in a 5 L sterile plastic container and was initiated by mixing collected fermented pulque and fresh aguamiel (3:2 v/v) (as recommended by the local pulque supplier). Aliquots for metagenomic DNA extraction were sampled immediately after mixing PQ and fresh AM (0 h, T0), at 3 h (T3), at 6 h (T6), and from AM and fermented pulque (PQ). The concentration of sucrose, glucose, fructose, ethanol, lactic-, and acetic acids were determined in all samples using a Waters HPLC system, equipped with an Aminex column for fermentation analysis as previously reported^23^.

### Illumina shotgun sequencing of pulque fermentation

Four 10 mL aliquot from each fermentation stage (AM, T0, T3, T6 and PQ) was centrifuged at 10,000×*g* for 40 min at 4° C to sediment the total cells present in each sample. The pellet was washed with 10 mL of 1× PBS buffer 3 times. Next, cells were successively lysed by resuspending the pellet in 9.5 mL TE buffer and 0.5 mL SDS 10% and adding (i) 5 mg crystalline lysozyme from chicken egg white (Sigma) for 40 min at 42° C, and (ii) by adding 3 µL of 20 mg/mL of proteinase K (Sigma) for 10 min at 60° C. Lysates were treated with 1.8 mL NaCl 5 M and 1.5 mL of CTAB/NaCl and incubated for 10 min at 65° C. DNA was extracted by adding one volume of chloroform/isoamyl alcohol (24:1). DNA recovered from the aqueous phase was purified via silica gel spin filtration (Collection Tube 2 of the MoBio kit). After this step, we followed the instructions in the MoBio Kit. (MoBio Cat. no. 12224-250). Finally, DNA resuspended with TRIS buffer pH 8.0.

Samples were sequenced on a MiSeq Illumina Sequencer (Instituto Nacional de Medicina Genómica, Mexico City) producing paired-end 145 bp reads. Four technical replicates were sequenced at each stage (AM, T0, T3, T6, and PQ). Raw sequencing data was improved as follows: first identical read pairs, which likely represent PCR duplicates, were collapsed using Tally^64^. Next, residual adapter sequences were trimmed from reads using Trim Galore v0.4.3 (https://www.bioinformatics.babraham.ac.uk/projects/trim_galore/) with the “--stringency = 1” parameter. Trim galore was also used to trim reads at bases with quality scores < Q30. Read pairs were discarded when one read was < 50 bp. Quality improved read sets were inspected via FASTQC (https://www.bioinformatics.babraham.ac.uk/projects/fastqc/). Raw metagenomics is available in the NCBI Sequence Read Archive under BioProject accession PRJNA603591.

### Bioinformatic analysis of microbial composition and functional profiles

Microbial composition and abundance for all stages and all replicates was predicted using MetaPhlan 2.0^28^ and Kaiju v1.7.3^29^. MetaPhlan relies on unique clade specific markers from a database of over 3,000 genomes to make taxonomic predictions at various levels. We used the BowTie2 --bt2_ps “very-sensitive” preset parameters, --tax_lev ‘a’ for prediction of all taxonomic levels, “--min_cu_len 2000” for minimum total nucleotide length for the markers in the clade, and “--stat_q 0,1” for quantile value. Kaiju was also used for taxonomic classification of shotgun metagenomics reads. Kaiju searches reads against the NCBI NR database and, because of the database size, has better resolution at the species level. Kaiju was run using the “greedy” algorithm against the NCBI BLAST nr + euk database which consists of over 100 million NCBI NR protein sequences from bacteria, archaea, viruses, fungi and other microbial eukaryotes.

Metagenomic data was also used to predict the functional profile of each stage and each replicate, using SUPER-FOCUS^30^. This tool reports functional annotation for 4 subsystem levels (levels 1–3 and seed function) using CD-HIT^65^. Briefly, SUPER-FOCUS identifies the taxonomic profile of the data and creates a database with the subsystems for predicted organism. Metagenomic data was aligned against the database using RAPSearch2^66^. Sequences with e-values ≤ 1e^−5^, a minimum identity of 60%, and an alignment length ≥ 15 amino acids were retained. Output with the subsystem levels ranges from general function (level 1) to a specific function (seed level). We identified level 2 functional groups that were significantly different between AM versus PQ, AM versus T0, T0 versus T3, T3 versus T6, and T6 versus PQ. We considered functional groups significantly different in abundance when (a) there was ≥ 1.5-fold difference in relative abundance between groups, and (b) Pearson's chi-squared test with absolute counts for each group yielded *p* values ≤ 0.000273 (Bonferroni corrected *p* value cutoff; *p* value = 0.05/183 level 2 categories). Pearson's chi-squared test was performed in JMP Pro 14.0.0. To identify the SUPERFOCUS subsystem presence and absence patterns across the major genera identified with Kaiju, we extracted organism to pathway information from the “organisms2subsystem.txt” file and linked pathway numbers to subsystems using the “database_PKs.txt” file, both of which were obtained from the “db” directory in the SUPERFOCUS package.

### Statistical analysis and data visualization

Statistical analyses were performed in R.3.2.2^67^ and JMP Pro 14.0.0. Principal component analysis (PCA) was performed using the *prcomp* function to compare species relative abundance and functional group relative abundance across all stages. Data visualization was performed using ggplot2^68^, Heatplus^69^, ggfortify^70^ and RColorBrewer^71^ packages.

### Evolutionary analysis of assembled *S. cerevisiae* metagenomics scaffolds from pulque

We used the cleaned and trimmed metagenomics read sets from the PQ fermentation stage to perform a metagenomics assembly using MetaSPAdes^33^, with a *k*-mer range of 33, 43, 53, 63 and 73. Contigs or scaffolds ≥ 2,000 bp were retained for subsequent analysis. Autometa^34^ and MetaBAT2^35^ binning algorithms were used to independently recover contigs and scaffolds identified as *S. cerevisiae*.

We analyzed *S. cerevisiae* contigs/scaffolds identified from the metagenomic data with 157 *S. cerevisiae* strains from Gallone et al.^31^ and the reference 288C genome. To eliminate bias in gene prediction stemming from different approaches, we performed gene prediction in all genome assemblies with Augustus^72^ using the *S. cerevisiae* training set. Translated coding sequence (CDS) files were used to identify orthologous groups with Orthofinder^37^ and to perform phylogenetic analysis. Orthofinder was run using diamond v0.8.22^73^ and distant matrices were inferred by dendroblast^74^. The species tree was inferred from unrooted orthogroup gene trees and a consensus tree was estimated using the STAG algorithm^38^. Tree visualization was performed using the ggtree^75^ R package.

### Whole-genome analysis of *Z. mobilis* isolates from pulque

We isolated three strains of *Z. mobilis* from the laboratory pulque fermentation described above. We modified a previously described method for *Zymomonas* enrichment and isolation^76^. Briefly, a 5 mL aliquot of pulque was taken after 3 h of fermentation, transferred to a 50 mL Falcon tube containing 30 mL of enrichment broth^76^ and incubated at 30° C for 5 h. Serial dilutions were performed and plated in agar Zm containing 3 g/L malt extract, 3 g/L yeast extract, 20 g/L glucose, 5 g/L peptone (all reagents from DIFCO) and 1 µg/mL of cycloheximide (Fluka). Plates were incubated overnight (ON) in an anaerobic jar at 30 °C. Colonies were transferred to a fresh agar Zm plate and incubated in an anaerobic jar at 30 °C ON. Colonies were verified visually by microscopy for purity and gram stain. Selected colonies were cultured in 3 mL of Zm broth (pH 6.8) at 30 °C, ON and screened for ethanol smell and gas production. Chromosomal DNA of selected colonies was extracted with the UltraClean Microbial DNA extraction kit (MoBio). 16S rDNA was PCR amplified and sequenced as described previously^23^ and resultant sequences were identified in the NCBI non-redundant database.

Library preparation and whole-genome paired-end 152 bp Illumina sequencing was conducted at Macrogen (Rockville, Maryland). Improvement of raw sequencing data was performed as follows: First identical read pairs, which likely represent PCR duplicates, were collapsed using Tally^64^. Next, residual adapter sequences were trimmed from reads using TrimGalore v0.4.3 (https://www.bioinformatics.babraham.ac.uk/projects/trim_galore/) with the “--stringency = 1” parameter. TrimGalore was also used to trim reads at bases with quality scores < Q30. Read pairs were discarded when one read was < 50 bp. Finally, error correction was performed with SPAdes v3.10^77^. We used the Unicycler pipeline^78^, which uses SPAdes v3.10, to assemble the *Z. mobilis* genomes. We used the “--careful” “-k 21,35,47,57,67,73,81,85,91,95” parameters in SPAdes for genome assembly. Raw whole-genome Illumina sequence data is available in the NCBI Sequence Read Archive under BioProject accession PRJNA603591.

### Evolutionary relationships of *Z. mobilis* isolates

We used the PhaME package^39^ to identify single nucleotide polymorphisms between seven publicly available *Z. mobilis* genomes and the three *Z. mobilis* genome we isolated from pulque. Strains extracted from the NCBI database included (strain (GenBank accession number)): ATCC31821 (ASM710v1), ATCC10988 (ASM17525v2), ATCC29191 (ASM27775v1), NRRL B-14023 (ASM57616v1), NRRL B-12526 (ASM57612v1), NRRL B-1960 (ASM215884v1), NCIMB 11163 (ASM2424v1)^40–45^. We used the ATCC31821 genome as the reference during PHaME analysis. The alignment consisted of 74,825 polymorphic sites. This data was used to construct a maximum likelihood phylogenetic tree using RaxML^79^. We implemented the generalized time reversible model (GTR) with 100 bootstrap replicates. Additionally, we used SplitTree4 to build a phylogenetic network in order to assess recombination between isolates^80^. We used the NeighborNet method with 100 bootstrap replicates.

### Measuring the relative abundance of sequenced *Z. mobilis* genotypes during fermentation

We predicted the relative proportion of the sequenced *Z. mobilis* isolates across each stage of fermentation. D5 the T32 isolates are very closely related and may represent a clonal lineage or the same isolate (Fig. 5). Thus, we grouped these isolates together. We performed two independent analyses to identify and quantify the relative abundance of the AM2 and D5/T32 genotypes. First, reads for each isolate were mapped against the reference *Z. mobilis* ATCC10988 genome^44^ using bwa mem v0.7.15^81^ with default settings. We chose this reference genome because it was isolate from pulque and closely related to our sequenced strains (Fig. 5). Next, sorted bam files were generated with samtools v1.4.1^82^ and read group information was added to each sorted bam file using the bamaddrg program (https://github.com/ekg/bamaddrg). We then used freebayes v1.3.1 to identify variants using the parameters “--ploidy 1” and “-C 40”^83^. Next, we used vcftools v0.1.14^84^ to filter our variant file with the following parameters “-remove-indels”, “--remove-filtered-all”, “--min-meanDP 20”, “--minQ 20”, “--recode” and “--recode-INFO-all”. Finally, we used GATK to convert the VCF file to table format^85^. Using these criteria, we identified 380 SNP that differentiated the AM2 and D5/T32 genotypes. Next, using the procedure above, we mapped metagenomics reads for each fermentation stage against the *Z. mobilis* ATCC10988 genome and used the samtools “mpileup” function to quantify allele frequency at each of the 380 polymorphic sites. We averaged the 380 allele frequencies for the AM2 and D5/T32 genotypes to quantify the relative proportion of each genotype.

Additionally, we used the LS-BSR pipeline^86^ with default settings to identify lineage-specific genes between the genome assemblies of AM2, D5, and ATCC10988. We used the ATCC10988 genome because it was the reference genome used for metagenomics mapping and genes uniquely shared by AM2 and ATCC10988 or D5 and ATCC10988 could have falsely inflated relative abundance estimates. We used the “compare_BSR.py” script included in LS-BSR software to detect genes that were unique to ATCC10988, AM2 and D5. We identified 24 genes unique to AM2 and 7 genes unique to D5. We collected all consensus genes between the three isolates and merged them into a single fasta file which was used as a mapping reference file for the metagenomics read sets. Using the approach above, we mapped read sets from each fermentation stage against the gene consensus fasta file, and then used the samtools “depth” function to calculate read depth across the 31 genes unique to either AM2 or D5. We averaged read depth values for each gene across fermentation stages and divided the AM2 average read depth by the sum of the averaged value of AM2 and D5 average read depths for each stage. This value gives the relative abundance estimate of the AM2 genotype.

## Supplementary information

Supplementary Information 1.

Supplementary Information 2.

## Data Availability

Raw Illumina shotgun metagenomics data from aguamiel and pulque fermentation and Raw whole-genome Illumina sequence data from three *Z. mobilis* strains isolated from pulque are available through the NCBI Sequence Read Archive under BioProject accession PRJNA603591.
