# Supplementary material for: Genomic profiling of bacterial and fungal communities and their predictive functionality during pulque fermentation by whole-genome shotgun sequencing
Source: Sci Rep. 2020 Sep 15;10:15115. doi: 10.1038/s41598-020-71864-4 (PMC7493934; doi:10.1038/s41598-020-71864-4)
Supplement: Supplementary file 1 — Supplementary Information 1. [file 41598_2020_71864_MOESM1_ESM.docx]

**Supplementary Information**

**Title:**

Genomic Profiling of Bacterial and Fungal communities and their Predictive Functionality during Pulque Fermentation by Whole-Genome Shotgun Sequencing

**Authors:**

Katherine Chacón-Vargas^1,2^, Julian Torres^3^, Martha Giles-Gómez^4^, Adelfo Escalante^4*^, John G. Gibbons^1,2,5*^

**Author Affiliations:**

^1^Molecular and Cellular Biology Graduate Program, University of Massachusetts, Amherst, MA 01003, USA.

^2^Department of Food Science, University of Massachusetts, Amherst, MA 01003, USA.

^3^Departamento de Ingeniería Celular y Biocatálisis, Instituto de Biotecnología, Universidad Nacional Autónoma de México, Cuernavaca, Mexico.

^4^Departamento de Biología, Facultad de Química, Universidad Nacional Autónoma de México, Ciudad de México, Mexico.

^5^Organismic & Evolutionary Biology Graduate Program, University of Massachusetts, Amherst, MA 01003, USA.


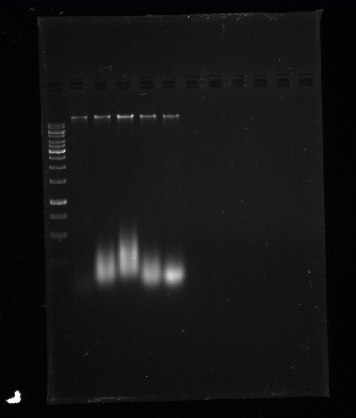


500 bp

10,000 bp

**Metagenomic DNA**

6,000 bp

3,000 bp

1,000 bp

1

2

3

4

5

6

**Supplementary Figure S1. Representative gel image (1%) of total DNA extraction from pulque fermentation.** High molecular weight DNA is observed above 10,000 kbps. Ribosomal RNA is observed at the bottom of the gel. Column 1: ladder; Column 2-6: samples (Aguamiel, Pulque, T_0hrs, T_3hrs and T_6hrs). Molecular weight ladder: GeneRuler 1 kb DNA Ladder, Thermo Fischer Scientific, #SM0311.


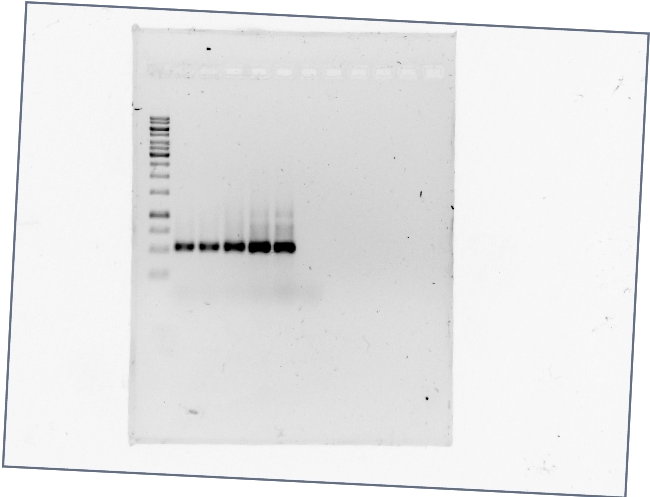


250 bps

500 bps

**~550 bps**

1000 bps

3000 bps

65º

63º

57º

56º

55º

H_2_O

Ladder

**Supplementary Figure S2. Representative gel image of annealing temperature optimization of 16S rDNA (V3-V4 region) from pulque metagenomics DNA extraction.** Validation of metagenomics DNA isolation for 16S rDNA (V3-V4) PCR amplification. Annealing temperature optimization (65C, 63C, 57C, 56C, and 55C) for the V3-V4 region. PCR reaction as follows: primer F1 (1mM) 8.8ul, primer R1 (1mM) 8.8ul, buffer 13.2 ul, dNTPs (10mM) 1.65 ul, H20 15.73ul, NZYTaq polymerase 1.32ul, DNA (25ng/ul) 1ul.

**Supplementary Figure S3. MetaPhlAn based Organismal diversity during pulque fermentation.** (A) Genus-level and (B) Species-level relative abundance during pulque fermentation. Columns represent relative abundance of organisms (Y-axis) per fermentation stage (X-axis). AM = Aguamiel, T0 = pulque and aguamiel mixture, T3 = 3-hours fermentation, T6 = 6-hours fermentation, and PQ = ~12-hours fermentation (mature pulque). Values are reported as percentages. Principal Component Analysis (PCA) of relative organismal abundance for genera (C) and species (D). In both comparisons, principal components 1 and 2 explain >94% of variance.


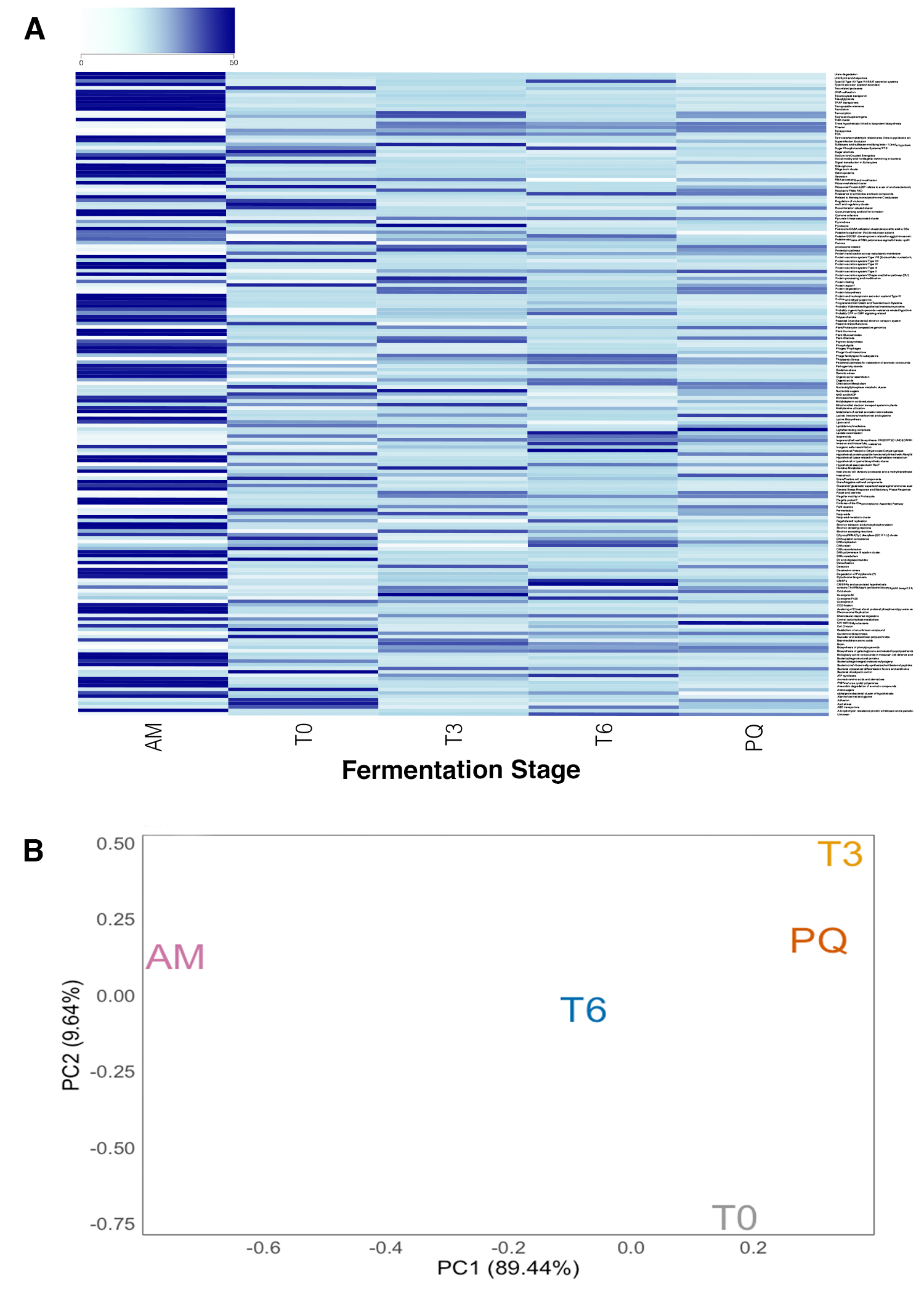


**Supplementary Figure S4. Metagenomic functional profiles change during pulque fermentation.** (A) Heatmap of relative abundance for level-2 functional groups as defined by SUPER-FOCUS (X-axis) during pulque fermentation (Y-axis), where dark blue indicates high abundance and light blue indicates low abundance. (B) Principal Component Analysis (PCA) of functional profiles. Principal components 1 and 2 explain >99% of variance.


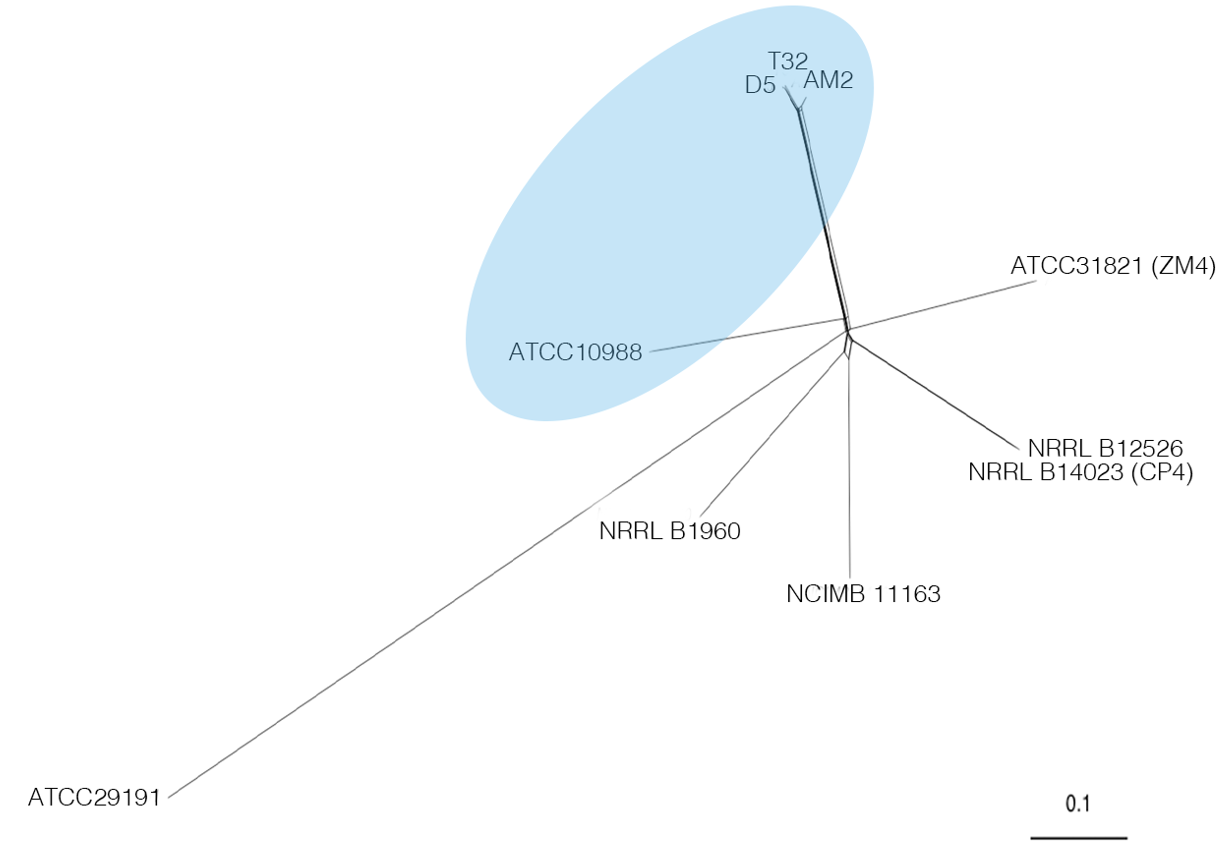


**Supplementary Figure S5. Phylogenetic Network Analysis of 10 *Zymomonas mobilis* genomes.**


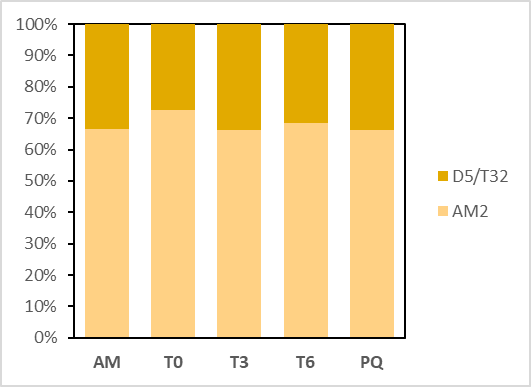

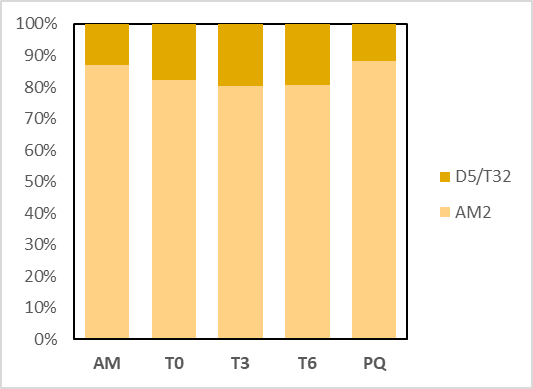


**Relative Abundance**

**Relative Abundance**

**A**

**B**

**Supplementary Figure S6. Relative abundance of AM2 and D5/T32 genotypes.** (A) Relative abundance of AM2 and D5/T32 genotypes estimated by differentiated SNPs. (B) Relative abundance of AM2 and D5/T32 genotypes estimated by the read depth of lineage-specific genes**.** Y-axis represents relative abundance of genotype. X-axis represents fermentation stage.

**Supplementary Table S1.** Genus-level organismal abundance estimated with MetaPhlAn across stages of pulque fermentation. AM = Aguamiel, T0 = seed preparation, T3 = 3-hours fermentation, T6 = 6-hours fermentation, PQ = 24-hours fermentation (mature pulque). Values are reported as percentages.

| **Genus** | **AM** | **T0** | **T3** | **T6** | **PQ** |
| --- | --- | --- | --- | --- | --- |
| ***Acetobacter*** | 1 | 1.3 | 1.26 | 1.89 | 1.16 |
| ***Acinetobacter*** | 50.52 | 10.84 | 9.97 | 13.19 | 5.89 |
| ***Enterobacteriaceae*** | 0.01 | 0 | 0 | 0 | 0 |
| ***Eremothecium*** | 0 | 0.1 | 0.28 | 0.07 | 0.19 |
| ***Escherichia*** | 0.01 | 0 | 0 | 0.01 | 0 |
| ***Gluconacetobacter*** | 0.06 | 0.08 | 0.11 | 0.14 | 0.14 |
| ***Lactobacillus*** | 0 | 0.65 | 0.54 | 1.09 | 1.37 |
| ***Lactococcus*** | 0.22 | 0.04 | 0.1 | 0.12 | 0.11 |
| ***Leuconostoc*** | 34.98 | 46.3 | 25.6 | 34.61 | 32.78 |
| ***Naumovozyma*** | 0.01 | 0.43 | 0.77 | 0.23 | 0.59 |
| ***Pseudomona*** | 0 | 0 | 0 | 0.05 | 0.02 |
| ***Rahnella*** | 0.15 | 0 | 0.02 | 0.02 | 0.01 |
| ***Saccharomyces*** | 0 | 3.94 | 5.36 | 1.61 | 4.42 |
| ***Zymomonas*** | 12.96 | 34.34 | 40.49 | 46.11 | 50.82 |
| **other** | 0.08 | 1.98 | 15.5 | 0.87 | 2.49 |

**Supplementary Table S2.** Genus-level organismal abundance estimated with Kaiju across stages of pulque fermentation. AM = Aguamiel, T0 = seed preparation, T3 = 3-hours fermentation, T6 = 6-hours fermentation, PQ = 24-hours fermentation (mature pulque). Values are reported as percentages.

| **Genus** | **AM** | **T0** | **T3** | **T6** | **PQ** |
| --- | --- | --- | --- | --- | --- |
| ***Acetobacter*** | 0.511963 | 0.533985 | 0.521273 | 0.646715 | 0.516535 |
| ***Acinetobacter*** | 21.94959 | 5.732172 | 5.446959 | 7.166111 | 2.845929 |
| ***Alkanindiges*** | 0.185028 | 0.046048 | 0.044156 | 0.059513 | 0.022663 |
| ***Bacillus*** | 0.220073 | 0.159816 | 0.113505 | 0.160282 | 0.143433 |
| ***Enterobacter*** | 0.114948 | 0.021941 | 0.020343 | 0.027412 | 0.014415 |
| ***Enterococcus*** | 0.263149 | 0.361923 | 0.216571 | 0.268122 | 0.263989 |
| ***Erwinia*** | 0.192985 | 0.046215 | 0.046383 | 0.052991 | 0.028957 |
| ***Escherichia*** | 0.172761 | 0.039996 | 0.040256 | 0.048838 | 0.026049 |
| ***Fructobacillus*** | 0.089492 | 0.115294 | 0.080439 | 0.111225 | 0.10404 |
| ***Gluconobacter*** | 0.256467 | 0.156059 | 0.171996 | 0.233597 | 0.144707 |
| ***Hafnia*** | 0.442518 | 0.158033 | 0.184493 | 0.179421 | 0.132225 |
| ***Klebsiella*** | 0.157801 | 0.020722 | 0.017319 | 0.024195 | 0.012255 |
| ***Kluyvera*** | 0.262349 | 0.084146 | 0.102805 | 0.122883 | 0.075608 |
| ***Kluyveromyces*** | 0.171449 | 0.231485 | 0.208352 | 0.246467 | 0.233476 |
| ***Komagataeibacter*** | 0.057626 | 0.081286 | 0.08825 | 0.116323 | 0.089482 |
| ***Lactobacillus*** | 0.965185 | 4.943326 | 3.608864 | 7.079878 | 7.533407 |
| ***Lactococcus*** | 13.71848 | 19.52196 | 11.25322 | 11.73195 | 13.03275 |
| ***Leuconostoc*** | 13.92343 | 17.34637 | 12.37795 | 16.49912 | 14.29545 |
| ***Moraxella*** | 0.17395 | 0.047638 | 0.043037 | 0.059966 | 0.025635 |
| ***Pediococcus*** | 0.036058 | 0.080991 | 0.055015 | 0.106818 | 0.101183 |
| ***Pseudomonas*** | 0.327991 | 0.108497 | 0.103359 | 0.148629 | 0.072568 |
| ***Psychrobacter*** | 0.138308 | 0.030468 | 0.027403 | 0.039753 | 0.015151 |
| ***Rahnella*** | 0.368188 | 0.098893 | 0.12442 | 0.141167 | 0.088248 |
| ***Saccharomyces*** | 0.033242 | 10.79021 | 19.07889 | 5.653661 | 13.51074 |
| ***Salmonella*** | 0.102228 | 0.015555 | 0.012627 | 0.019545 | 0.009251 |
| ***Serratia*** | 0.128244 | 0.037264 | 0.041064 | 0.048342 | 0.028925 |
| ***Sphingomonas*** | 0.042592 | 0.069604 | 0.107395 | 0.122563 | 0.109757 |
| ***Staphylococcus*** | 0.115706 | 0.139876 | 0.097927 | 0.139105 | 0.130554 |
| ***Streptococcus*** | 0.585461 | 0.933761 | 0.539389 | 0.60869 | 0.639657 |
| ***Weissella*** | 0.19811 | 0.190745 | 0.131039 | 0.187241 | 0.168448 |
| ***Zymomonas*** | 4.77526 | 12.56924 | 19.90855 | 22.26569 | 21.48354 |

**Supplementary Table S3.** Species-level organismal abundance estimated with MetaPhlAn across stages of pulque fermentation. AM = Aguamiel, T0 = seed preparation, T3 = 3-hours fermentation, T6 = 6-hours fermentation, PQ = 24-hours fermentation (mature pulque). Values are reported as percentages.

| **Species** | **AM** | **T0** | **T3** | **T6** | **PQ** |
| --- | --- | --- | --- | --- | --- |
| ***Acetobacter aceti*** | 0 | 0 | 0 | 0 | 0 |
| ***Acetobacter pasteurianus*** | 0.33 | 0.22 | 0.18 | 0.19 | 0.2 |
| ***Acetobacter unclassified*** | 0.68 | 1.08 | 1.08 | 1.7 | 0.95 |
| ***Acinetobacter unclassified*** | 50.52 | 10.84 | 9.97 | 13.19 | 5.89 |
| ***Enterobacteriaceae bacterium 9_2_54FAA*** | 0.01 | 0 | 0 | 0 | 0 |
| ***Eremothecium unclassified*** | 0 | 0.1 | 0.28 | 0.07 | 0.19 |
| ***Escherichia unclassified*** | 0.01 | 0 | 0 | 0.01 | 0 |
| ***Gluconacetobacter unclassified*** | 0.06 | 0.08 | 0.11 | 0.14 | 0.14 |
| ***Lactobacillus brevis*** | 0 | 0 | 0 | 0.03 | 0.02 |
| ***Lactobacillus hilgardii*** | 0 | 0 | 0.04 | 0.13 | 0.09 |
| ***Lactobacillus otakiensis*** | 0 | 0.16 | 0.21 | 0.48 | 0.38 |
| ***Lactobacillus sanfranciscensis*** | 0 | 0.48 | 0.28 | 0.43 | 0.88 |
| ***Lactococcus lactis*** | 0.22 | 0.04 | 0.1 | 0.12 | 0.11 |
| ***Leuconostoc citreum*** | 0.33 | 0.25 | 0.1 | 0.14 | 0.15 |
| ***Leuconostoc gelidum*** | 0 | 0 | 0 | 0 | 0 |
| ***Leuconostoc inhae*** | 0 | 0 | 0 | 0 | 0 |
| ***Leuconostoc kimchii*** | 1.01 | 1.19 | 0.7 | 0.93 | 0.89 |
| ***Leuconostoc mesenteroides*** | 0.72 | 0.37 | 0.22 | 0.28 | 0.33 |
| ***Leuconostoc unclassified*** | 32.92 | 44.5 | 24.58 | 33.26 | 31.42 |
| ***Naumovozyma unclassified*** | 0.01 | 0.43 | 0.77 | 0.23 | 0.59 |
| ***Pseudomonas unclassified*** | 0 | 0 | 0 | 0.05 | 0.02 |
| ***Rahnella aquatilis*** | 0.02 | 0 | 0 | 0 | 0.01 |
| ***Rahnella unclassified*** | 0.13 | 0 | 0.02 | 0.02 | 0.01 |
| ***Saccharomyces cerevisiae*** | 0 | 3.94 | 5.36 | 1.61 | 4.42 |
| ***Zymomonas mobilis*** | 12.96 | 34.34 | 40.49 | 46.11 | 50.82 |
| **other** | 0.08 | 1.98 | 15.5 | 0.87 | 2.49 |

**Supplementary Table S4.** Species-level organismal abundance estimated with Kaiju across stages of pulque fermentation. AM = Aguamiel, T0 = seed preparation, T3 = 3-hours fermentation, T6 = 6-hours fermentation, PQ = 24-hours fermentation (mature pulque). Values are reported as percentages.

| **Species** | **AM** | **T0** | **T3** | **T6** | **PQ** |
| --- | --- | --- | --- | --- | --- |
| ***Acinetobacter baumannii*** | 0.640346 | 0.141581 | 0.136024 | 0.176323 | 0.069361 |
| ***Acinetobacter boissieri*** | 1.588875 | 0.442337 | 0.414991 | 0.538942 | 0.216312 |
| ***Acinetobacter brisouii*** | 0.439452 | 0.123885 | 0.112827 | 0.151708 | 0.059896 |
| ***Acinetobacter celticus*** | 0.103284 | 0.027352 | 0.027001 | 0.035437 | 0.013878 |
| ***Acinetobacter equi*** | 0.206051 | 0.059038 | 0.05541 | 0.072006 | 0.028945 |
| ***Acinetobacter gerneri*** | 0.309618 | 0.076196 | 0.074261 | 0.098994 | 0.038832 |
| ***Acinetobacter haemolyticus*** | 0.145908 | 0.017093 | 0.013283 | 0.025684 | 0.007122 |
| ***Acinetobacter harbinensis*** | 0.112484 | 0.031802 | 0.03232 | 0.041152 | 0.016468 |
| ***Acinetobacter junii*** | 0.106298 | 0.01299 | 0.010711 | 0.017497 | 0.005686 |
| ***Acinetobacter larvae*** | 0.291 | 0.080414 | 0.077782 | 0.100956 | 0.041789 |
| ***Acinetobacter nectaris*** | 2.684998 | 0.74776 | 0.708451 | 0.929459 | 0.373265 |
| ***Acinetobacter pittii*** | 0.180569 | 0.027814 | 0.024073 | 0.036932 | 0.013086 |
| ***Acinetobacter qingfengensis*** | 0.343634 | 0.097174 | 0.095774 | 0.120577 | 0.049193 |
| ***Acinetobacter rudis*** | 0.267256 | 0.073464 | 0.06966 | 0.089928 | 0.03621 |
| ***Acinetobacter seohaensis*** | 0.227128 | 0.063 | 0.059514 | 0.075634 | 0.031042 |
| ***Acinetobacter sp. ANC 3789*** | 0.213118 | 0.057076 | 0.056151 | 0.071868 | 0.029558 |
| ***Acinetobacter sp. HR7*** | 0.103556 | 0.02707 | 0.02643 | 0.035748 | 0.014081 |
| ***Acinetobacter sp. NCu2D-2*** | 0.128665 | 0.035995 | 0.035729 | 0.045272 | 0.018879 |
| ***Acinetobacter sp. P8-3-8*** | 0.122719 | 0.034507 | 0.034276 | 0.043209 | 0.01788 |
| ***Acinetobacter sp. TGL-Y2*** | 0.155006 | 0.045035 | 0.041505 | 0.054715 | 0.022229 |
| ***Acinetobacter sp. WCHA30*** | 0.176361 | 0.045779 | 0.045259 | 0.059952 | 0.024593 |
| ***Acinetobacter sp. WCHA34*** | 0.122511 | 0.033738 | 0.031614 | 0.041324 | 0.0165 |
| ***Acinetobacter sp. WCHA60*** | 0.100506 | 0.026788 | 0.026996 | 0.035103 | 0.01383 |
| ***Acinetobacter tandoii*** | 0.217273 | 0.05941 | 0.058157 | 0.077487 | 0.03027 |
| ***Acinetobacter towneri*** | 0.181614 | 0.042009 | 0.035373 | 0.048308 | 0.017948 |
| ***Escherichia coli*** | 0.163636 | 0.037457 | 0.037566 | 0.045157 | 0.024115 |
| ***Hafnia alvei*** | 0.418412 | 0.147544 | 0.172527 | 0.165075 | 0.124881 |
| ***Klebsiella pneumoniae*** | 0.111994 | 0.011926 | 0.007919 | 0.011988 | 0.005916 |
| ***Kluyvera intermedia*** | 0.200718 | 0.064385 | 0.07854 | 0.093957 | 0.057242 |
| ***Kluyveromyces marxianus*** | 0.155433 | 0.209775 | 0.184782 | 0.223872 | 0.210189 |
| ***Lactobacillus florum*** | 0.003451 | 0.069386 | 0.040301 | 0.063857 | 0.10622 |
| ***Lactobacillus lindneri*** | 0.011845 | 0.253772 | 0.157226 | 0.247985 | 0.417166 |
| ***Lactobacillus otakiensis*** | 0.001413 | 0.047625 | 0.065584 | 0.13603 | 0.09463 |
| ***Lactobacillus plantarum*** | 0.040453 | 0.110998 | 0.087793 | 0.202293 | 0.136602 |
| ***Lactobacillus sanfranciscensis*** | 0.084154 | 2.216506 | 1.412629 | 2.198799 | 3.709124 |
| ***Lactococcus chungangensis*** | 0.316125 | 0.417781 | 0.227457 | 0.245995 | 0.265549 |
| ***Lactococcus lactis*** | 0.969868 | 1.111914 | 0.690256 | 0.761606 | 0.795265 |
| ***Lactococcus piscium*** | 1.646741 | 2.440875 | 1.315923 | 1.388074 | 1.575265 |
| ***Lactococcus plantarum*** | 8.504792 | 12.10883 | 7.075361 | 7.33354 | 8.116817 |
| ***Lactococcus raffinolactis*** | 0.55248 | 0.822212 | 0.442066 | 0.462911 | 0.528722 |
| ***Leuconostoc carnosum*** | 0.270632 | 0.372079 | 0.272297 | 0.355334 | 0.310302 |
| ***Leuconostoc citreum*** | 1.681104 | 1.254431 | 0.87276 | 1.19898 | 1.039349 |
| ***Leuconostoc fallax*** | 0.10153 | 0.121372 | 0.079732 | 0.108036 | 0.096596 |
| ***Leuconostoc gelidum*** | 1.765946 | 1.748228 | 1.285315 | 1.722731 | 1.470612 |
| ***Leuconostoc kimchii*** | 0.273021 | 0.301576 | 0.216407 | 0.292617 | 0.255005 |
| ***Leuconostoc lactis*** | 0.105855 | 0.111049 | 0.081145 | 0.108379 | 0.093743 |
| ***Leuconostoc mesenteroides*** | 0.896626 | 0.863182 | 0.589944 | 0.828766 | 0.722781 |
| ***Leuconostoc sp. C2*** | 0.18123 | 0.262299 | 0.177739 | 0.237244 | 0.208382 |
| ***Saccharomyces arboricola*** | 0.001707 | 0.432514 | 0.792479 | 0.230154 | 0.559434 |
| ***Saccharomyces cerevisiae*** | 0.006379 | 2.415228 | 4.049596 | 1.246453 | 2.855633 |
| ***S. cerevisiae x S. kudriavzevii*** | 0.000789 | 0.264594 | 0.462613 | 0.13668 | 0.326121 |
| ***Saccharomyces eubayanus*** | 0.003061 | 0.55245 | 0.98895 | 0.285213 | 0.698964 |
| ***Saccharomyces kudriavzevii*** | 0.000635 | 0.194682 | 0.367528 | 0.104933 | 0.256748 |
| ***Salmonella enterica*** | 0.101034 | 0.015106 | 0.012034 | 0.018647 | 0.008785 |
| ***Streptococcus pneumoniae*** | 0.063631 | 0.125129 | 0.070231 | 0.082108 | 0.078345 |
| ***Zymomonas mobilis*** | 4.77526 | 12.56924 | 19.90855 | 22.26569 | 21.48354 |

**Supplementary Table S5.** Pearson correlations (*r*) between chemical abundance and organismal abundance across pulque fermentation.

| **Chemical** | **Species** | **Correlation (*r*)** | **P-value** |
| --- | --- | --- | --- |
| *Acetate* | *Acinetobacter baumannii* | -0.4135 | 0.4889 |
| *Acetate* | *Acinetobacter boissieri* | -0.3774 | 0.5312 |
| *Acetate* | *Acinetobacter brisouii* | -0.3722 | 0.5373 |
| *Acetate* | *Acinetobacter equi* | -0.3706 | 0.5391 |
| *Acetate* | *Acinetobacter gerneri* | -0.3832 | 0.5243 |
| *Acetate* | *Acinetobacter larvae* | -0.3724 | 0.537 |
| *Acetate* | *Acinetobacter nectaris* | -0.3713 | 0.5384 |
| *Acetate* | *Acinetobacter qingfengensis* | -0.3688 | 0.5414 |
| *Acetate* | *Acinetobacter rudis* | -0.3789 | 0.5294 |
| *Acetate* | *Acinetobacter seohaensis* | -0.3842 | 0.5231 |
| *Acetate* | *Acinetobacter sp. ANC 3789* | -0.3777 | 0.5308 |
| *Acetate* | *Acinetobacter tandoii* | -0.3577 | 0.5545 |
| *Acetate* | *Hafnia alvei* | -0.4411 | 0.4571 |
| *Acetate* | *Kluyvera intermedia* | -0.3238 | 0.5951 |
| *Acetate* | *Kluyveromyces marxianus* | 0.6266 | 0.2581 |
| *Acetate* | *Lactobacillus lindneri* | 0.1671 | 0.7882 |
| *Acetate* | *Lactobacillus plantarum* | 0.8002 | 0.1039 |
| *Acetate* | *Lactobacillus sanfranciscensis* | 0.1717 | 0.7825 |
| *Acetate* | *Lactococcus chungangensis* | -0.4425 | 0.4556 |
| *Acetate* | *Lactococcus lactis* | -0.5002 | 0.3908 |
| *Acetate* | *Lactococcus piscium* | -0.3857 | 0.5213 |
| *Acetate* | *Lactococcus plantarum* | -0.3802 | 0.5279 |
| *Acetate* | *Lactococcus raffinolactis* | -0.3902 | 0.5161 |
| *Acetate* | *Leuconostoc carnosum* | 0.4378 | 0.461 |
| *Acetate* | *Leuconostoc citreum* | -0.3886 | 0.5179 |
| *Acetate* | *Leuconostoc gelidum* | -0.0157 | 0.9801 |
| *Acetate* | *Leuconostoc kimchii* | 0.1375 | 0.8255 |
| *Acetate* | *Leuconostoc mesenteroides* | -0.1504 | 0.8092 |
| *Acetate* | *Leuconostoc sp. C2* | 0.3303 | 0.5872 |
| *Acetate* | *Saccharomyces arboricola* | 0.0911 | 0.8841 |
| *Acetate* | *Saccharomyces cerevisiae* | 0.1002 | 0.8726 |
| *Acetate* | *Saccharomyces cerevisiae x Saccharomyces kudriavzevii* | 0.0919 | 0.8831 |
| *Acetate* | *Saccharomyces eubayanus* | 0.0855 | 0.8913 |
| *Acetate* | *Saccharomyces kudriavzevii* | 0.0924 | 0.8825 |
| *Acetate* | *Zymomonas mobilis* | 0.6559 | 0.2293 |
| *Ethanol* | *Acinetobacter baumannii* | -0.9302 | 0.0219 |
| *Ethanol* | *Acinetobacter boissieri* | -0.9321 | 0.021 |
| *Ethanol* | *Acinetobacter brisouii* | -0.9301 | 0.022 |
| *Ethanol* | *Acinetobacter equi* | -0.9318 | 0.0212 |
| *Ethanol* | *Acinetobacter gerneri* | -0.9262 | 0.0238 |
| *Ethanol* | *Acinetobacter larvae* | -0.9287 | 0.0226 |
| *Ethanol* | *Acinetobacter nectaris* | -0.9297 | 0.0221 |
| *Ethanol* | *Acinetobacter qingfengensis* | -0.9317 | 0.0212 |
| *Ethanol* | *Acinetobacter rudis* | -0.9319 | 0.0211 |
| *Ethanol* | *Acinetobacter seohaensis* | -0.934 | 0.0202 |
| *Ethanol* | *Acinetobacter sp. ANC 3789* | -0.9295 | 0.0222 |
| *Ethanol* | *Acinetobacter tandoii* | -0.925 | 0.0244 |
| *Ethanol* | *Hafnia alvei* | -0.9332 | 0.0205 |
| *Ethanol* | *Kluyvera intermedia* | -0.8892 | 0.0435 |
| *Ethanol* | *Kluyveromyces marxianus* | 0.8806 | 0.0486 |
| *Ethanol* | *Lactobacillus lindneri* | 0.9027 | 0.0359 |
| *Ethanol* | *Lactobacillus plantarum* | 0.8168 | 0.0915 |
| *Ethanol* | *Lactobacillus sanfranciscensis* | 0.9071 | 0.0335 |
| *Ethanol* | *Lactococcus chungangensis* | -0.3626 | 0.5486 |
| *Ethanol* | *Lactococcus lactis* | -0.4976 | 0.3937 |
| *Ethanol* | *Lactococcus piscium* | -0.1844 | 0.7665 |
| *Ethanol* | *Lactococcus plantarum* | -0.1917 | 0.7575 |
| *Ethanol* | *Lactococcus raffinolactis* | -0.1852 | 0.7655 |
| *Ethanol* | *Leuconostoc carnosum* | 0.4561 | 0.4401 |
| *Ethanol* | *Leuconostoc citreum* | -0.8068 | 0.099 |
| *Ethanol* | *Leuconostoc gelidum* | -0.4231 | 0.4778 |
| *Ethanol* | *Leuconostoc kimchii* | -0.0939 | 0.8806 |
| *Ethanol* | *Leuconostoc mesenteroides* | -0.4804 | 0.4127 |
| *Ethanol* | *Leuconostoc sp. C2* | 0.3819 | 0.5258 |
| *Ethanol* | *Saccharomyces arboricola* | 0.6025 | 0.2823 |
| *Ethanol* | *Saccharomyces cerevisiae* | 0.6083 | 0.2764 |
| *Ethanol* | *Saccharomyces cerevisiae x Saccharomyces kudriavzevii* | 0.603 | 0.2817 |
| *Ethanol* | *Saccharomyces eubayanus* | 0.5998 | 0.285 |
| *Ethanol* | *Saccharomyces kudriavzevii* | 0.5978 | 0.287 |
| *Ethanol* | *Zymomonas mobilis* | 0.9479 | 0.0141 |
| *Fructose* | *Acinetobacter baumannii* | -0.8967 | 0.0392 |
| *Fructose* | *Acinetobacter boissieri* | -0.8839 | 0.0466 |
| *Fructose* | *Acinetobacter brisouii* | -0.8831 | 0.0471 |
| *Fructose* | *Acinetobacter equi* | -0.8811 | 0.0483 |
| *Fructose* | *Acinetobacter gerneri* | -0.884 | 0.0466 |
| *Fructose* | *Acinetobacter larvae* | -0.8802 | 0.0489 |
| *Fructose* | *Acinetobacter nectaris* | -0.8808 | 0.0485 |
| *Fructose* | *Acinetobacter qingfengensis* | -0.8777 | 0.0504 |
| *Fructose* | *Acinetobacter rudis* | -0.884 | 0.0466 |
| *Fructose* | *Acinetobacter seohaensis* | -0.8865 | 0.0451 |
| *Fructose* | *Acinetobacter sp. ANC 3789* | -0.8815 | 0.0481 |
| *Fructose* | *Acinetobacter tandoii* | -0.8731 | 0.0532 |
| *Fructose* | *Hafnia alvei* | -0.8887 | 0.0438 |
| *Fructose* | *Kluyvera intermedia* | -0.8358 | 0.0779 |
| *Fructose* | *Kluyveromyces marxianus* | 0.7918 | 0.1104 |
| *Fructose* | *Lactobacillus lindneri* | 0.6494 | 0.2356 |
| *Fructose* | *Lactobacillus plantarum* | 0.7716 | 0.1264 |
| *Fructose* | *Lactobacillus sanfranciscensis* | 0.6563 | 0.229 |
| *Fructose* | *Lactococcus chungangensis* | -0.4717 | 0.4225 |
| *Fructose* | *Lactococcus lactis* | -0.6212 | 0.2634 |
| *Fructose* | *Lactococcus piscium* | -0.3059 | 0.6167 |
| *Fructose* | *Lactococcus plantarum* | -0.3038 | 0.6192 |
| *Fructose* | *Lactococcus raffinolactis* | -0.3061 | 0.6165 |
| *Fructose* | *Leuconostoc carnosum* | 0.3779 | 0.5306 |
| *Fructose* | *Leuconostoc citreum* | -0.8876 | 0.0445 |
| *Fructose* | *Leuconostoc gelidum* | -0.5201 | 0.369 |
| *Fructose* | *Leuconostoc kimchii* | -0.2239 | 0.7173 |
| *Fructose* | *Leuconostoc mesenteroides* | -0.6164 | 0.2682 |
| *Fructose* | *Leuconostoc sp. C2* | 0.2896 | 0.6364 |
| *Fructose* | *Saccharomyces arboricola* | 0.6832 | 0.2035 |
| *Fructose* | *Saccharomyces cerevisiae* | 0.6899 | 0.1973 |
| *Fructose* | *Saccharomyces cerevisiae x Saccharomyces kudriavzevii* | 0.6839 | 0.2029 |
| *Fructose* | *Saccharomyces eubayanus* | 0.6795 | 0.207 |
| *Fructose* | *Saccharomyces kudriavzevii* | 0.6817 | 0.205 |
| *Fructose* | *Zymomonas mobilis* | 0.9578 | 0.0103 |
| *Glucose* | *Acinetobacter baumannii* | -0.4586 | 0.4373 |
| *Glucose* | *Acinetobacter boissieri* | -0.4399 | 0.4586 |
| *Glucose* | *Acinetobacter brisouii* | -0.4451 | 0.4526 |
| *Glucose* | *Acinetobacter equi* | -0.4363 | 0.4627 |
| *Glucose* | *Acinetobacter gerneri* | -0.4471 | 0.4503 |
| *Glucose* | *Acinetobacter larvae* | -0.4379 | 0.4608 |
| *Glucose* | *Acinetobacter nectaris* | -0.4387 | 0.4599 |
| *Glucose* | *Acinetobacter qingfengensis* | -0.4268 | 0.4736 |
| *Glucose* | *Acinetobacter rudis* | -0.4392 | 0.4593 |
| *Glucose* | *Acinetobacter seohaensis* | -0.4395 | 0.459 |
| *Glucose* | *Acinetobacter sp. ANC 3789* | -0.4367 | 0.4621 |
| *Glucose* | *Acinetobacter tandoii* | -0.4329 | 0.4666 |
| *Glucose* | *Hafnia alvei* | -0.4076 | 0.4958 |
| *Glucose* | *Kluyvera intermedia* | -0.4073 | 0.4961 |
| *Glucose* | *Kluyveromyces marxianus* | 0.1181 | 0.85 |
| *Glucose* | *Lactobacillus lindneri* | -0.064 | 0.9186 |
| *Glucose* | *Lactobacillus plantarum* | 0.1142 | 0.8549 |
| *Glucose* | *Lactobacillus sanfranciscensis* | -0.0544 | 0.9308 |
| *Glucose* | *Lactococcus chungangensis* | -0.4603 | 0.4354 |
| *Glucose* | *Lactococcus lactis* | -0.5796 | 0.3057 |
| *Glucose* | *Lactococcus piscium* | -0.3775 | 0.531 |
| *Glucose* | *Lactococcus plantarum* | -0.3599 | 0.5518 |
| *Glucose* | *Lactococcus raffinolactis* | -0.3737 | 0.5355 |
| *Glucose* | *Leuconostoc carnosum* | -0.1497 | 0.8101 |
| *Glucose* | *Leuconostoc citreum* | -0.7306 | 0.1609 |
| *Glucose* | *Leuconostoc gelidum* | -0.6895 | 0.1978 |
| *Glucose* | *Leuconostoc kimchii* | -0.6019 | 0.2828 |
| *Glucose* | *Leuconostoc mesenteroides* | -0.7734 | 0.125 |
| *Glucose* | *Leuconostoc sp. C2* | -0.1999 | 0.7471 |
| *Glucose* | *Saccharomyces arboricola* | 0.7157 | 0.174 |
| *Glucose* | *Saccharomyces cerevisiae* | 0.7132 | 0.1763 |
| *Glucose* | *Saccharomyces cerevisiae x Saccharomyces kudriavzevii* | 0.7136 | 0.1759 |
| *Glucose* | *Saccharomyces eubayanus* | 0.7122 | 0.1772 |
| *Glucose* | *Saccharomyces kudriavzevii* | 0.7214 | 0.169 |
| *Glucose* | *Zymomonas mobilis* | 0.5012 | 0.3896 |
| *Lactate* | *Acinetobacter baumannii* | -0.7965 | 0.1068 |
| *Lactate* | *Acinetobacter boissieri* | -0.7962 | 0.107 |
| *Lactate* | *Acinetobacter brisouii* | -0.7929 | 0.1096 |
| *Lactate* | *Acinetobacter equi* | -0.7954 | 0.1076 |
| *Lactate* | *Acinetobacter gerneri* | -0.7875 | 0.1138 |
| *Lactate* | *Acinetobacter larvae* | -0.7905 | 0.1114 |
| *Lactate* | *Acinetobacter nectaris* | -0.7921 | 0.1102 |
| *Lactate* | *Acinetobacter qingfengensis* | -0.7948 | 0.1081 |
| *Lactate* | *Acinetobacter rudis* | -0.7961 | 0.1071 |
| *Lactate* | *Acinetobacter seohaensis* | -0.7996 | 0.1044 |
| *Lactate* | *Acinetobacter sp. ANC 3789* | -0.792 | 0.1102 |
| *Lactate* | *Acinetobacter tandoii* | -0.7838 | 0.1167 |
| *Lactate* | *Hafnia alvei* | -0.8067 | 0.099 |
| *Lactate* | *Kluyvera intermedia* | -0.7296 | 0.1618 |
| *Lactate* | *Kluyveromyces marxianus* | 0.8594 | 0.0619 |
| *Lactate* | *Lactobacillus lindneri* | 0.83 | 0.0819 |
| *Lactate* | *Lactobacillus plantarum* | 0.8987 | 0.0381 |
| *Lactate* | *Lactobacillus sanfranciscensis* | 0.8354 | 0.0781 |
| *Lactate* | *Lactococcus chungangensis* | -0.496 | 0.3955 |
| *Lactate* | *Lactococcus lactis* | -0.5887 | 0.2963 |
| *Lactate* | *Lactococcus piscium* | -0.3495 | 0.5642 |
| *Lactate* | *Lactococcus plantarum* | -0.3588 | 0.5531 |
| *Lactate* | *Lactococcus raffinolactis* | -0.3521 | 0.5611 |
| *Lactate* | *Leuconostoc carnosum* | 0.4184 | 0.4833 |
| *Lactate* | *Leuconostoc citreum* | -0.682 | 0.2047 |
| *Lactate* | *Leuconostoc gelidum* | -0.3165 | 0.6039 |
| *Lactate* | *Leuconostoc kimchii* | -0.0339 | 0.9569 |
| *Lactate* | *Leuconostoc mesenteroides* | -0.3787 | 0.5296 |
| *Lactate* | *Leuconostoc sp. C2* | 0.3227 | 0.5964 |
| *Lactate* | *Saccharomyces arboricola* | 0.4096 | 0.4935 |
| *Lactate* | *Saccharomyces cerevisiae* | 0.4077 | 0.4957 |
| *Lactate* | *Saccharomyces cerevisiae x Saccharomyces kudriavzevii* | 0.4054 | 0.4983 |
| *Lactate* | *Saccharomyces eubayanus* | 0.4039 | 0.5001 |
| *Lactate* | *Saccharomyces kudriavzevii* | 0.4072 | 0.4962 |
| *Lactate* | *Zymomonas mobilis* | 0.9475 | 0.0143 |
| *Sucrose* | *Acinetobacter baumannii* | 0.9146 | 0.0296 |
| *Sucrose* | *Acinetobacter boissieri* | 0.9125 | 0.0306 |
| *Sucrose* | *Acinetobacter brisouii* | 0.9128 | 0.0305 |
| *Sucrose* | *Acinetobacter equi* | 0.9115 | 0.0312 |
| *Sucrose* | *Acinetobacter gerneri* | 0.9077 | 0.0332 |
| *Sucrose* | *Acinetobacter larvae* | 0.908 | 0.033 |
| *Sucrose* | *Acinetobacter nectaris* | 0.9098 | 0.0321 |
| *Sucrose* | *Acinetobacter qingfengensis* | 0.9075 | 0.0333 |
| *Sucrose* | *Acinetobacter rudis* | 0.9119 | 0.031 |
| *Sucrose* | *Acinetobacter seohaensis* | 0.914 | 0.0299 |
| *Sucrose* | *Acinetobacter sp. ANC 3789* | 0.9079 | 0.0331 |
| *Sucrose* | *Acinetobacter tandoii* | 0.903 | 0.0357 |
| *Sucrose* | *Hafnia alvei* | 0.8966 | 0.0393 |
| *Sucrose* | *Kluyvera intermedia* | 0.8542 | 0.0654 |
| *Sucrose* | *Kluyveromyces marxianus* | -0.7616 | 0.1346 |
| *Sucrose* | *Lactobacillus lindneri* | -0.7431 | 0.1501 |
| *Sucrose* | *Lactobacillus plantarum* | -0.7334 | 0.1584 |
| *Sucrose* | *Lactobacillus sanfranciscensis* | -0.751 | 0.1434 |
| *Sucrose* | *Lactococcus chungangensis* | 0.5341 | 0.3538 |
| *Sucrose* | *Lactococcus lactis* | 0.6763 | 0.21 |
| *Sucrose* | *Lactococcus piscium* | 0.3612 | 0.5503 |
| *Sucrose* | *Lactococcus plantarum* | 0.3626 | 0.5486 |
| *Sucrose* | *Lactococcus raffinolactis* | 0.3608 | 0.5508 |
| *Sucrose* | *Leuconostoc carnosum* | -0.2778 | 0.6509 |
| *Sucrose* | *Leuconostoc citreum* | 0.9205 | 0.0266 |
| *Sucrose* | *Leuconostoc gelidum* | 0.6116 | 0.273 |
| *Sucrose* | *Leuconostoc kimchii* | 0.3191 | 0.6008 |
| *Sucrose* | *Leuconostoc mesenteroides* | 0.6828 | 0.2039 |
| *Sucrose* | *Leuconostoc sp. C2* | -0.192 | 0.757 |
| *Sucrose* | *Saccharomyces arboricola* | -0.73 | 0.1614 |
| *Sucrose* | *Saccharomyces cerevisiae* | -0.7289 | 0.1624 |
| *Sucrose* | *Saccharomyces cerevisiae x Saccharomyces kudriavzevii* | -0.727 | 0.1641 |
| *Sucrose* | *Saccharomyces eubayanus* | -0.7253 | 0.1655 |
| *Sucrose* | *Saccharomyces kudriavzevii* | -0.7291 | 0.1622 |
| *Sucrose* | *Zymomonas mobilis* | -0.9877 | 0.0016 |

**Supplementary Table S6.** Superfocus level-2 functional groups with significantly different abundance between aguamiel (AM) and pulque (PQ). *P*-values < 1e^-300^ are represented as 1e^-300^.

| **Level 2** | **AM Relative Abundance** | **PQ Relative Abundance** | **PQ/AM Relative Abundance** | **P-value** |
| --- | --- | --- | --- | --- |
| Bacteriocins, ribosomally synthesized antibacterial peptides | 0.266 | 0.177 | 0.667 | 1.00E-300 |
| Carotenoid biosynthesis | 0.071 | 0.275 | 3.897 | 1.00E-300 |
| clustering of 2 heat shock proteins, phosphoenolpyruvate carboxykinase and a putative hydrolase | 0.048 | 0.008 | 0.163 | 1.00E-300 |
| CO2 fixation | 0.307 | 0.154 | 0.502 | 1.00E-300 |
| Detection | 0.009 | 0.041 | 4.437 | 1.00E-300 |
| Detoxification | 0.349 | 0.244 | 0.699 | 1.00E-300 |
| DNA polymerase III epsilon cluster | 0.096 | 0.042 | 0.434 | 1.00E-300 |
| Electron donating reactions | 1.096 | 0.490 | 0.448 | 1.00E-300 |
| Fe-S clusters | 0.176 | 0.296 | 1.680 | 1.00E-300 |
| Flagellar motility in Prokaryota | 0.465 | 1.098 | 2.359 | 1.00E-300 |
| Folate and pterines | 1.364 | 2.065 | 1.514 | 1.00E-300 |
| General Stress Response and Stationary Phase Response | 0.077 | 0.010 | 0.126 | 1.00E-300 |
| Gram-Negative cell wall components | 1.589 | 0.591 | 0.372 | 1.00E-300 |
| Metabolism of central aromatic intermediates | 0.190 | 0.053 | 0.277 | 1.00E-300 |
| Organic sulfur assimilation | 0.600 | 0.448 | 0.748 | 1.00E-300 |
| Osmotic stress | 0.678 | 0.490 | 0.723 | 1.00E-300 |
| Pathogenicity islands | 0.121 | 0.057 | 0.469 | 1.00E-300 |
| Phages, Prophages | 1.168 | 0.663 | 0.568 | 1.00E-300 |
| Plant Alkaloids | 0.003 | 0.032 | 9.421 | 1.00E-300 |
| Probably Ybbk-related hypothetical membrane proteins | 0.025 | 0.003 | 0.131 | 1.00E-300 |
| Programmed Cell Death and Toxin-antitoxin Systems | 0.337 | 0.183 | 0.544 | 1.00E-300 |
| Proline and 4-hydroxyproline | 0.335 | 0.188 | 0.560 | 1.00E-300 |
| Protein and nucleoprotein secretion system, Type IV | 0.681 | 0.159 | 0.234 | 1.00E-300 |
| Protein secretion system, Type VI | 0.171 | 0.043 | 0.255 | 1.00E-300 |
| Proteolytic pathway | 0.001 | 0.042 | 58.862 | 1.00E-300 |
| Quinone cofactors | 0.994 | 0.519 | 0.522 | 1.00E-300 |
| Quorum sensing and biofilm formation | 0.131 | 0.023 | 0.174 | 1.00E-300 |
| Ribosome-related cluster | 0.053 | 0.008 | 0.145 | 1.00E-300 |
| Secretion | 0.044 | 0.007 | 0.148 | 1.00E-300 |
| Selenoproteins | 0.120 | 0.046 | 0.381 | 1.00E-300 |
| Siderophores | 0.114 | 0.024 | 0.208 | 1.00E-300 |
| Social motility and nonflagellar swimming in bacteria | 0.230 | 0.038 | 0.164 | 1.00E-300 |
| Translation | 0.215 | 0.089 | 0.416 | 1.00E-300 |
| Triacylglycerols | 0.051 | 0.013 | 0.243 | 1.00E-300 |
| Uni- Sym- and Antiporters | 0.276 | 0.114 | 0.411 | 1.00E-300 |
| Urate degradation | 0.035 | 0.004 | 0.108 | 1.00E-300 |
| Inorganic sulfur assimilation | 0.223 | 0.144 | 0.644 | 1.20E-296 |
| Transposable elements | 0.103 | 0.054 | 0.522 | 2.40E-270 |
| Biosynthesis of phenylpropanoids | 0.010 | 0.039 | 4.022 | 1.40E-261 |
| heat shock, cell division, proteases, and a methyltransferase | 0.028 | 0.006 | 0.230 | 4.70E-249 |
| Lysine Biosynthesis | 0.019 | 0.003 | 0.164 | 6.30E-220 |
| DNA metabolism | 0.028 | 0.008 | 0.272 | 2.10E-217 |
| Bacteriophage integration/excision/lysogeny | 0.076 | 0.039 | 0.511 | 2.00E-211 |
| Invasion and intracellular resistance | 0.018 | 0.048 | 2.647 | 4.80E-208 |
| Electron transport and photophosphorylation | 0.010 | 0.000 | 0.047 | 8.60E-170 |
| Thiamin | 0.019 | 0.045 | 2.366 | 3.00E-162 |
| Isoprenoid/cell wall biosynthesis: PREDICTED UNDECAPRENYL DIPHOSPHATE PHOSPHATASE | 0.062 | 0.102 | 1.640 | 8.00E-154 |
| CRISPs | 0.044 | 0.075 | 1.699 | 1.40E-127 |
| Fatty acid metabolic cluster | 0.044 | 0.022 | 0.506 | 4.40E-125 |
| Hypothetical in Lysine biosynthetic cluster | 0.016 | 0.005 | 0.291 | 1.80E-117 |
| D-tyrosyl-tRNA(Tyr) deacylase (EC 3.1.-.-) cluster | 0.043 | 0.023 | 0.529 | 1.50E-111 |
| Cold shock | 0.036 | 0.062 | 1.709 | 1.20E-107 |
| proteosome related | 0.007 | 0.021 | 2.984 | 4.30E-105 |
| tRNA sulfuration | 0.035 | 0.017 | 0.495 | 1.10E-104 |
| Polysaccharides | 0.039 | 0.021 | 0.536 | 2.34E-95 |
| Lactate racemization | 0.001 | 0.009 | 8.120 | 3.92E-87 |
| Regulation of virulence | 0.036 | 0.020 | 0.559 | 3.94E-79 |
| Bacteriophage structural proteins | 0.022 | 0.010 | 0.466 | 8.36E-77 |
| Three hypotheticals linked to lipoprotein biosynthesis | 0.004 | 0.013 | 3.187 | 5.53E-73 |
| Plastidial (cyanobacterial) electron transport system | 0.004 | 0.000 | 0.005 | 1.86E-72 |
| Cytochrome biogenesis | 0.109 | 0.081 | 0.744 | 5.19E-70 |
| Hypothetical lipase related to Phosphatidate metabolism | 0.005 | 0.001 | 0.114 | 1.94E-63 |
| Fimbriae of the Chaperone/Usher Assembly Pathway | 0.006 | 0.001 | 0.217 | 1.81E-59 |
| Bacterial cytostatics, differentiation factors and antibiotics | 0.003 | 0.009 | 3.554 | 2.61E-57 |
| Toxins and superantigens | 0.000 | 0.005 | 11.480 | 5.08E-56 |
| A bicyclomycin resistance protein, a helicase, and a pseudouridine synthase | 0.005 | 0.001 | 0.215 | 1.78E-47 |
| Type III, Type IV, Type VI, ESAT secretion systems | 0.019 | 0.010 | 0.557 | 9.64E-42 |
| Chromosome Replication | 0.006 | 0.002 | 0.339 | 1.68E-39 |
| Protein secretion system, Chaperone-Usher pathway (CU) | 0.003 | 0.001 | 0.229 | 6.51E-32 |
| Putrescine/GABA utilization cluster-temporal,to add to SSs | 0.008 | 0.004 | 0.465 | 1.74E-30 |
| Protein secretion system, Type III | 0.003 | 0.001 | 0.249 | 1.03E-29 |
| Tartronate-semialdehyde related area (links to pyridoxine and aldorate metabolism) | 0.002 | 0.000 | 0.144 | 2.99E-23 |
| Shiga toxin cluster | 0.002 | 0.000 | 0.214 | 3.12E-21 |
| Flagella protein? | 0.002 | 0.001 | 0.263 | 3.19E-20 |
| Pyruvate kinase associated cluster | 0.001 | 0.002 | 3.023 | 4.47E-14 |
| Protein secretion system, Type VIII (Extracellular nucleation/precipitation pathway, ENP) | 0.002 | 0.001 | 0.305 | 1.05E-12 |
| Hypothetical associated with RecF | 0.006 | 0.009 | 1.532 | 5.89E-12 |
| Anaerobic degradation of aromatic compounds | 0.001 | 0.000 | 0.202 | 3.36E-11 |
| Dessication stress | 0.001 | 0.000 | 0.213 | 3.14E-07 |
| Tricarboxylate transporter | 0.001 | 0.001 | 0.440 | 8.40E-07 |
| Type III secretion system, extended | 0.000 | 0.000 | 0.164 | 4.33E-06 |
| Chemotaxis, response regulators | 0.001 | 0.002 | 1.759 | 6.98E-06 |
| Biologically active compounds in metazoan cell defence and differentiation | 0.000 | 0.000 | 0.318 | 0.000126 |
| alpha-proteobacterial cluster of hypotheticals | 0.003 | 0.002 | 0.674 | 0.000172 |
| CRISPRs and associated hypotheticals | 0.001 | 0.002 | 1.549 | 0.000265 |

**Supplementary Table S7.** Superfocus level-2 functional groups with significantly different abundance between aguamiel (AM) and seed-preparation stage (T0). *P*-values < 1e^-300^ are represented as 1e^-300^.

| **Level 2** | **AM Relative Abundance** | **T0 Relative Abundance** | **T0/AM Relative Abundance** | ***P-value*** |
| --- | --- | --- | --- | --- |
| Carotenoid biosynthesis | 0.071 | 0.172 | 2.433 | 1.00E-300 |
| Electron donating reactions | 1.096 | 0.543 | 0.496 | 1.00E-300 |
| Flagellar motility in Prokaryota | 0.465 | 0.715 | 1.535 | 1.00E-300 |
| Gram-Negative cell wall components | 1.589 | 0.709 | 0.446 | 1.00E-300 |
| Metabolism of central aromatic intermediates | 0.190 | 0.072 | 0.379 | 1.00E-300 |
| Phages, Prophages | 1.168 | 0.651 | 0.557 | 1.00E-300 |
| Protein and nucleoprotein secretion system, Type IV | 0.681 | 0.248 | 0.364 | 1.00E-300 |
| Protein secretion system, Type VI | 0.171 | 0.058 | 0.342 | 1.00E-300 |
| Proteolytic pathway | 0.001 | 0.027 | 37.630 | 1.00E-300 |
| Quinone cofactors | 0.994 | 0.559 | 0.563 | 1.00E-300 |
| Quorum sensing and biofilm formation | 0.131 | 0.039 | 0.301 | 1.00E-300 |
| Social motility and nonflagellar swimming in bacteria | 0.230 | 0.062 | 0.268 | 1.00E-300 |
| Uni- Sym- and Antiporters | 0.276 | 0.127 | 0.459 | 1.00E-300 |
| Inorganic sulfur assimilation | 0.223 | 0.111 | 0.495 | 2.70E-291 |
| Siderophores | 0.114 | 0.040 | 0.351 | 9.50E-259 |
| Proline and 4-hydroxyproline | 0.335 | 0.209 | 0.624 | 3.80E-232 |
| Translation | 0.215 | 0.115 | 0.538 | 9.00E-232 |
| General Stress Response and Stationary Phase Response | 0.077 | 0.021 | 0.272 | 2.00E-228 |
| DNA polymerase III epsilon cluster | 0.096 | 0.036 | 0.379 | 5.00E-199 |
| Plant Alkaloids | 0.003 | 0.198 | 57.553 | 7.80E-170 |
| Ribosome-related cluster | 0.053 | 0.013 | 0.253 | 4.10E-167 |
| Programmed Cell Death and Toxin-antitoxin Systems | 0.337 | 0.230 | 0.684 | 5.70E-161 |
| Selenoproteins | 0.120 | 0.059 | 0.492 | 2.60E-160 |
| Invasion and intracellular resistance | 0.018 | 0.198 | 10.877 | 3.00E-150 |
| Protein translocation across cytoplasmic membrane | 0.130 | 0.198 | 1.518 | 2.10E-132 |
| Signal transduction in Eukaryotes | 0.106 | 0.198 | 1.857 | 1.90E-131 |
| clustering of 2 heat shock proteins, phosphoenolpyruvate carboxykinase and a putative hydrolase | 0.048 | 0.014 | 0.295 | 2.20E-131 |
| Detoxification | 0.349 | 0.253 | 0.725 | 5.40E-126 |
| Transposable elements | 0.103 | 0.053 | 0.513 | 1.00E-125 |
| CO2 fixation | 0.307 | 0.218 | 0.710 | 7.50E-124 |
| Triacylglycerols | 0.051 | 0.017 | 0.339 | 5.80E-123 |
| Biosynthesis of phenylpropanoids | 0.010 | 0.198 | 20.510 | 1.60E-108 |
| Detection | 0.009 | 0.198 | 21.137 | 2.50E-107 |
| Urate degradation | 0.035 | 0.009 | 0.270 | 2.10E-105 |
| Secretion | 0.044 | 0.016 | 0.355 | 1.10E-100 |
| Cytochrome biogenesis | 0.109 | 0.066 | 0.605 | 1.29E-84 |
| Probably Ybbk-related hypothetical membrane proteins | 0.025 | 0.007 | 0.304 | 4.44E-67 |
| Lysine Biosynthesis | 0.019 | 0.005 | 0.252 | 6.25E-62 |
| Electron transport and photophosphorylation | 0.010 | 0.000 | 0.032 | 1.21E-58 |
| Putative asociate of RNA polymerase sigma-54 factor rpoN | 0.061 | 0.035 | 0.577 | 5.24E-56 |
| DNA metabolism | 0.028 | 0.011 | 0.398 | 1.69E-55 |
| Pathogenicity islands | 0.121 | 0.084 | 0.696 | 7.45E-55 |
| heat shock, cell division, proteases, and a methyltransferase | 0.028 | 0.011 | 0.405 | 4.75E-54 |
| Toxins and superantigens | 0.000 | 0.004 | 10.562 | 2.56E-44 |
| Hypothetical in Lysine biosynthetic cluster | 0.016 | 0.005 | 0.312 | 9.57E-44 |
| Fatty acid metabolic cluster | 0.044 | 0.025 | 0.579 | 3.23E-40 |
| Lactate racemization | 0.001 | 0.005 | 5.148 | 1.63E-36 |
| Bacteriophage structural proteins | 0.022 | 0.011 | 0.483 | 6.88E-32 |
| Sugar Phosphotransferase Systems, PTS | 0.027 | 0.198 | 7.318 | 3.72E-28 |
| Plastidial (cyanobacterial) electron transport system | 0.004 | 0.000 | 0.030 | 1.52E-22 |
| proteosome related | 0.007 | 0.198 | 28.426 | 1.07E-21 |
| Fimbriae of the Chaperone/Usher Assembly Pathway | 0.006 | 0.002 | 0.265 | 3.14E-20 |
| Polysaccharides | 0.039 | 0.026 | 0.682 | 4.74E-20 |
| Bacterial checkpoint control | 0.014 | 0.023 | 1.589 | 5.79E-20 |
| tRNA sulfuration | 0.035 | 0.024 | 0.684 | 2.32E-18 |
| A bicyclomycin resistance protein, a helicase, and a pseudouridine synthase | 0.005 | 0.001 | 0.248 | 3.28E-17 |
| Hypothetical lipase related to Phosphatidate metabolism | 0.005 | 0.001 | 0.257 | 8.62E-16 |
| Putrescine/GABA utilization cluster-temporal,to add to SSs | 0.008 | 0.004 | 0.441 | 2.88E-15 |
| Type III, Type IV, Type VI, ESAT secretion systems | 0.019 | 0.012 | 0.627 | 7.04E-14 |
| Chromosome Replication | 0.006 | 0.003 | 0.419 | 5.69E-13 |
| Probably organic hydroperoxide resistance related hypothetical protein | 0.035 | 0.026 | 0.742 | 1.48E-12 |
| Protein secretion system, Type III | 0.003 | 0.001 | 0.269 | 1.18E-11 |
| Protein secretion system, Chaperone-Usher pathway (CU) | 0.003 | 0.001 | 0.301 | 9.92E-11 |
| Three hypotheticals linked to lipoprotein biosynthesis | 0.004 | 0.198 | 47.943 | 1.37E-09 |
| Hypothetical associated with RecF | 0.006 | 0.009 | 1.604 | 5.39E-09 |
| Shiga toxin cluster | 0.002 | 0.001 | 0.247 | 7.08E-08 |
| Tartronate-semialdehyde related area (links to pyridoxine and aldorate metabolism) | 0.002 | 0.000 | 0.217 | 1.06E-07 |
| Flagella protein? | 0.002 | 0.001 | 0.376 | 2.05E-06 |
| Protein secretion system, Type VIII (Extracellular nucleation/precipitation pathway, ENP) | 0.002 | 0.000 | 0.268 | 2.37E-06 |
| Bacterial cytostatics, differentiation factors and antibiotics | 0.003 | 0.198 | 75.985 | 2.45E-05 |
| Tricarboxylate transporter | 0.001 | 0.000 | 0.291 | 2.81E-05 |

**Supplementary Table S8.** Superfocus level-2 functional groups with significantly different abundance between seed-preparation stage (T0) and 3-hours fermentation (T3). *P*-values < 1e^-300^ are represented as 1e^-300^.

| **Level2** | **T0 Relative Abundance** | **T3 Relative Abundance** | **T0/T3 Relative Abundance** | ***P*-value** |
| --- | --- | --- | --- | --- |
| Flagellar motility in Prokaryota | 0.715 | 1.102 | 1.541 | 1.00E-300 |
| Gram-Positive cell wall components | 1.311 | 0.940 | 0.717 | 1.00E-300 |
| Carotenoid biosynthesis | 0.172 | 0.260 | 1.513 | 1.2E-152 |
| Ribosomal Protein L28P relates to a set of uncharacterized proteins | 0.233 | 0.164 | 0.706 | 1.5E-119 |
| Inorganic sulfur assimilation | 0.111 | 0.170 | 1.538 | 1.6E-106 |
| Proteolytic pathway | 0.027 | 0.060 | 2.237 | 3.2E-101 |
| DNA recombination | 0.184 | 0.130 | 0.710 | 1.82E-91 |
| Protein secretion system, Type VII | 0.098 | 0.062 | 0.636 | 3.52E-84 |
| Regulation of virulence | 0.039 | 0.020 | 0.524 | 3.4E-64 |
| Acid stress | 0.064 | 0.040 | 0.619 | 1.29E-61 |
| Bacteriophage integration/excision/lysogeny | 0.064 | 0.040 | 0.630 | 5.88E-57 |
| Pathogenicity islands | 0.084 | 0.060 | 0.713 | 5.24E-42 |
| Detection | 0.029 | 0.048 | 1.659 | 1.28E-40 |
| Putative asociate of RNA polymerase sigma-54 factor rpoN | 0.035 | 0.055 | 1.567 | 1.71E-38 |
| Thiamin | 0.027 | 0.044 | 1.617 | 1.51E-34 |
| Bacterial checkpoint control | 0.023 | 0.012 | 0.552 | 7.58E-33 |
| recX and regulatory cluster | 0.062 | 0.044 | 0.720 | 1.41E-29 |
| D-tyrosyl-tRNA(Tyr) deacylase (EC 3.1.-.-) cluster | 0.036 | 0.025 | 0.679 | 5.65E-24 |
| Plant Alkaloids | 0.023 | 0.034 | 1.508 | 4.08E-21 |
| Bacterial cytostatics, differentiation factors and antibiotics | 0.004 | 0.010 | 2.407 | 7.98E-21 |
| Plasmid related functions | 0.005 | 0.002 | 0.351 | 6.92E-19 |
| Nucleotidyl-phosphate metabolic cluster | 0.048 | 0.036 | 0.749 | 1.29E-18 |
| proteosome related | 0.013 | 0.021 | 1.608 | 3.34E-17 |
| Three hypotheticals linked to lipoprotein biosynthesis | 0.007 | 0.013 | 1.848 | 4.15E-16 |
| Catabolism of an unknown compound | 0.007 | 0.004 | 0.549 | 3.65E-11 |
| Pyruvate kinase associated cluster | 0.001 | 0.003 | 2.585 | 5.942E-07 |
| Nucleotide sugars | 0.001 | 0.002 | 3.033 | 0.000023 |

**Supplementary Table S9.** Superfocus level-2 functional groups with significantly different abundance between 3-hours fermentation (T3) and 6-hours fermentation (T6). *P*-values < 1e^-300^ are represented as 1e^-300^.

| **Level2** | **T3 Relative Abundance** | **T6 Relative Abundance** | **T6/T3 Relative Abundance** | ***P-value*** |
| --- | --- | --- | --- | --- |
| Proteolytic pathway | 0.060 | 0.016 | 0.257 | 1.00E-300 |
| Invasion and intracellular resistance | 0.039 | 0.073 | 1.845 | 2.20E-175 |
| Lactate racemization | 0.007 | 0.018 | 2.709 | 4.95E-93 |
| Mitochondrial electron transport system in plants | 0.016 | 0.007 | 0.454 | 6.81E-60 |
| Detection | 0.048 | 0.032 | 0.674 | 4.60E-55 |
| Plant Alkaloids | 0.034 | 0.021 | 0.626 | 2.29E-53 |
| Toxins and superantigens | 0.006 | 0.003 | 0.396 | 5.80E-31 |
| Polysaccharides | 0.027 | 0.019 | 0.719 | 5.70E-23 |
| CRISPRs and associated hypotheticals | 0.002 | 0.005 | 2.278 | 2.57E-19 |
| proteosome related | 0.021 | 0.016 | 0.740 | 7.34E-17 |
| Nucleotide sugars | 0.002 | 0.000 | 0.294 | 2.77E-12 |
| Plasmid related functions | 0.002 | 0.003 | 1.750 | 1.80E-07 |

**Supplementary Table S10.** Superfocus level-2 functional groups with significantly different abundance between 6-hours fermentation (T6) and 24-hours fermentation (PQ). *P*-values < 1e^-300^ are represented as 1e^-300^.

| **Level 2** | **AM Relative Abundance** | **PQ Relative Abundance** | **PQ/AM Relative Abundance** | **P-value** |
| --- | --- | --- | --- | --- |
| Gram-Negative cell wall components | 0.792 | 0.591 | 0.747 | 1.00E-300 |
| Protein and nucleoprotein secretion system, Type IV | 0.263 | 0.159 | 0.606 | 1.00E-300 |
| Social motility and nonflagellar swimming in bacteria | 0.080 | 0.038 | 0.474 | 8.20E-287 |
| Proteolytic pathway | 0.016 | 0.042 | 2.726 | 1.40E-227 |
| Programmed Cell Death and Toxin-antitoxin Systems | 0.254 | 0.183 | 0.722 | 8.50E-219 |
| Quorum sensing and biofilm formation | 0.043 | 0.023 | 0.528 | 8.60E-123 |
| Secretion | 0.018 | 0.007 | 0.354 | 1.00E-112 |
| Metabolism of central aromatic intermediates | 0.079 | 0.053 | 0.664 | 1.10E-103 |
| General Stress Response and Stationary Phase Response | 0.023 | 0.010 | 0.425 | 2.80E-103 |
| Invasion and intracellular resistance | 0.073 | 0.048 | 0.661 | 3.50E-97 |
| Type III, Type IV, Type VI, ESAT secretion systems | 0.021 | 0.010 | 0.499 | 8.11E-68 |
| Lactate racemization | 0.018 | 0.009 | 0.479 | 1.17E-65 |
| Ribosome-related cluster | 0.016 | 0.008 | 0.468 | 8.02E-64 |
| Siderophores | 0.037 | 0.024 | 0.645 | 5.63E-56 |
| Protein secretion system, Type VI | 0.060 | 0.043 | 0.725 | 1.87E-51 |
| clustering of 2 heat shock proteins, phosphoenolpyruvate carboxykinase and a putative hydrolase | 0.015 | 0.008 | 0.507 | 1.95E-49 |
| Plant Alkaloids | 0.021 | 0.032 | 1.522 | 1.52E-44 |
| Urate degradation | 0.009 | 0.004 | 0.421 | 7.29E-43 |
| Triacylglycerols | 0.020 | 0.013 | 0.627 | 1.90E-34 |
| Lysine Biosynthesis | 0.007 | 0.003 | 0.453 | 1.59E-29 |
| Probably Ybbk-related hypothetical membrane proteins | 0.007 | 0.003 | 0.477 | 6.52E-26 |
| DNA metabolism | 0.012 | 0.008 | 0.648 | 2.24E-19 |
| heat shock, cell division, proteases, and a methyltransferase | 0.010 | 0.006 | 0.621 | 6.14E-19 |
| CRISPRs and associated hypotheticals | 0.005 | 0.002 | 0.482 | 1.59E-17 |
| Toxins and superantigens | 0.003 | 0.005 | 1.954 | 4.21E-16 |
| Hypothetical in Lysine biosynthetic cluster | 0.007 | 0.005 | 0.644 | 1.03E-12 |
| Hypothetical lipase related to Phosphatidate metabolism | 0.002 | 0.001 | 0.331 | 8.14E-12 |
| Nucleotide sugars | 0.000 | 0.001 | 2.028 | 0.000241 |
